# Retinal Angiogenesis Regulates Astrocytic Differentiation in Neonatal Mouse Retinas by Oxygen Dependent Mechanisms

**DOI:** 10.1038/s41598-017-17962-2

**Published:** 2017-12-14

**Authors:** Li-Juan Duan, Sarah J. Pan, Thomas N. Sato, Guo-Hua Fong

**Affiliations:** 1 Center for Vascular Biology, University of Connecticut Health Center, 263 Farmington Ave, Farmington, CT 06032 USA; 2 Department of Cell Biology, University of Connecticut Health Center, 263 Farmington Ave, Farmington, CT 06032 USA; 3 The Thomas N. Sato BioMEC-X Laboratories, Advanced Telecommunications Research Institute International (ATR), ERATO Sato Live Bio-Forecasting Project, Japan Science and Technology Agency (JST), 619-0288 Kyoto, Japan

## Abstract

In mice, retinal vascular and astrocyte networks begin to develop at birth, expanding radially from the optic nerve head (ONH) towards the retinal periphery. The retinal vasculature grows towards the periphery ahead of differentiated astrocytes, but behind astrocytic progenitor cells (APCs) and immature astrocytes. Endothelial cell specific *Vegfr-2* disruption in newborn mice not only blocked retinal vascular development but also suppressed astrocytic differentiation, reducing the abundance of differentiated astrocytes while causing the accumulation of precursors. By contrast, retinal astrocytic differentiation was accelerated by the exposure of wild-type newborn mice to hyperoxia for 24 hours, or by APC specific deficiency in hypoxia inducible factor (HIF)−2α, an oxygen labile transcription factor. These findings reveal a novel function of the retinal vasculature, and imply that in normal neonatal mice, oxygen from the retinal circulation may promote astrocytic differentiation, in part by triggering oxygen dependent HIF-2α degradation in astrocytic precursors.

## Introduction

Blood vessels are traditionally known to support tissue metabolism. However, recent studies have shown that they also regulate cell differentiation in several organs including the liver, pancreas, lung, and the heart^1–3^. Endothelial cell (EC)-derived soluble protein factors known as “angiocrines” mediate these regulatory functions by interacting with their cognate receptors on target cells. However, it remains unclear whether tissue oxygenation by blood vessels also regulates cell differentiation. The main objective of this study is to address this issue, specifically focusing on how retinal astrocytic differentiation in neonatal mice is affected by retinal angiogenesis and tissue oxygenation.

Detailed studies in rats demonstrated that retinal astrocytes were derived from astrocytic progenitor cells (APCs) in the optic nerve, and that GFAP^+^ astrocytes populated the central 35% of the inner retinal surface by the time they were born^4–6^. A mixture of APCs and GFAP^+^ astrocytes were present in the retinas of newborn rats, but it was unclear whether both types of cells migrated out of the ONH independent of each other or only APCs migrated out of the ONH and then differentiated into astrocytes. By P5, rat retinas had no more APCs. Furthermore, only a small number of immature astrocytes (IACs) remained and were located at the leading front of the expanding astrocyte network. Mature astrocytes (mASCs, interchangeably referred to as differentiated astrocytes) were abundant and continued to spread, reaching the peripheral margin by P9^5^.

In mice, our knowledge about the normal morphological events of retinal astrocytic development is less detailed, although more is known at the molecular level^7–11^. While one study suggested that GFAP^+^ cells started migrating out of the ONH at post-conceptual day 17 and reached the retinal periphery by post-conceptual day 28 (equivalent to P8)^12^, another study concluded that GFAP^+^ astrocytes became detectable in the inner retinal surface only after P1, and reached the periphery by P10^13^. While these inconsistencies might be minor, a more fundamental but yet unresolved issue is when and where astrocytic differentiation occurs. Do astrocytic progenitors differentiate into astrocytes before they migrate out of the ONH and onto the inner retinal surface, or do they enter the retina initially as undifferentiated progenitors and then undergo differentiation?

Investigation of retinal astrocytic differentiation relies on the ability to identify different differentiation states, which is in turn influenced by the choice of astrocytic markers and the specific criteria employed to evaluate and interpret the relevant information. For the purpose of this study, Pax2 (paired box 2) and GFAP (glial fibrillary acidic protein) are two preferred markers for several reasons: (1) within neonatal retinas, Pax2 expression is specific to astrocytes and their precursors, undetectable in various other retinal cell types^6,14^; (2) GFAP expression is absent in undifferentiated Pax2^+^ progenitors, weak in immature astrocytes, but robustly upregulated in mature astrocytes, thus providing the basis for establishing quantitative criteria defining astrocytic differentiation states^14^. Although GFAP is also expressed in pathologically activated Müller cells in adult mice, the same has not been found in early neonates which are the focus of this study^5,13–16^; (3) Intracellularly, Pax2 expression is restricted to the nucleus. Thus, retinal astrocytes may be enumerated based on the number of Pax2^+^ nuclei, which would be otherwise difficult due to the lack of clear intercellular boundaries between stellate astrocytes.

Besides Pax2 and GFAP, other astrocytic markers also exist, such as vimentin and NG2^15,17,18^. However, the expression patterns of these markers are not consistently restricted to astrocytes. For example, vimentin is expressed in both astrocytes and Müller cells in neonatal mouse retinas^19^. After astrocytic maturation, vimentin expression diminishes in rats but persists in rabbits, beginning to decline only after P9^5,20^. Its regulation during mouse retinal development has not been characterized in detail. NG2 expression is not restricted to astrocytes or their progenitors, but also includes pericytes^21,22^. On the other hand, PDGFR-α is specifically expressed in immature and differentiated astrocytes, thus making it an appropriate marker to label and isolate essentially all retinal astrocytes regardless their differentiation status^17^.

It is believed that the retinal astrocyte network forms ahead of the vascular development and serves as a template to guide the radial progression of retinal angiogenesis from the ONH towards the retinal periphery^18,23–27^. The first observation leading to this astrocyte template theory was that possums which lacked retinal vasculature were also free of retinal astrocytes^23^. Subsequent studies observed that GFAP^+^ networks expanded towards the retinal periphery ahead of the vascular plexus^7,28^. Furthermore, several knockout mouse lines exhibited developmental defects in both retinal astrocytic and vascular networks, such as Tlx^−/−^ mice or neuroretina-selective *Hif-1α* knockout mice^8–11^. While the astrocyte template theory is well accepted, some authors noticed that ahead of the vascular plexus was immature astrocytes (IACs) instead of a network of differentiated astrocytes^5^.

In this study we found that astrocytic progenitors but not differentiated astrocytes migrated out of the ONH and into the retina, continuing to migrate towards the retinal periphery subsequently and differentiating into immature astrocytes along the way. Astrocytic differentiation was dependent on angiogenesis, and was accelerated by oxygen or by APC specific HIF-2α deficiency. Taken together, these findings suggest that blood vessels promote retinal astrocytic differentiation by oxygenating APCs and IACs, triggering oxygen dependent HIF-2α degradation in astrocytic precursors.

## Results

### APCs were abundantly present in the inner retinal surface at P0

**Figure 1:**
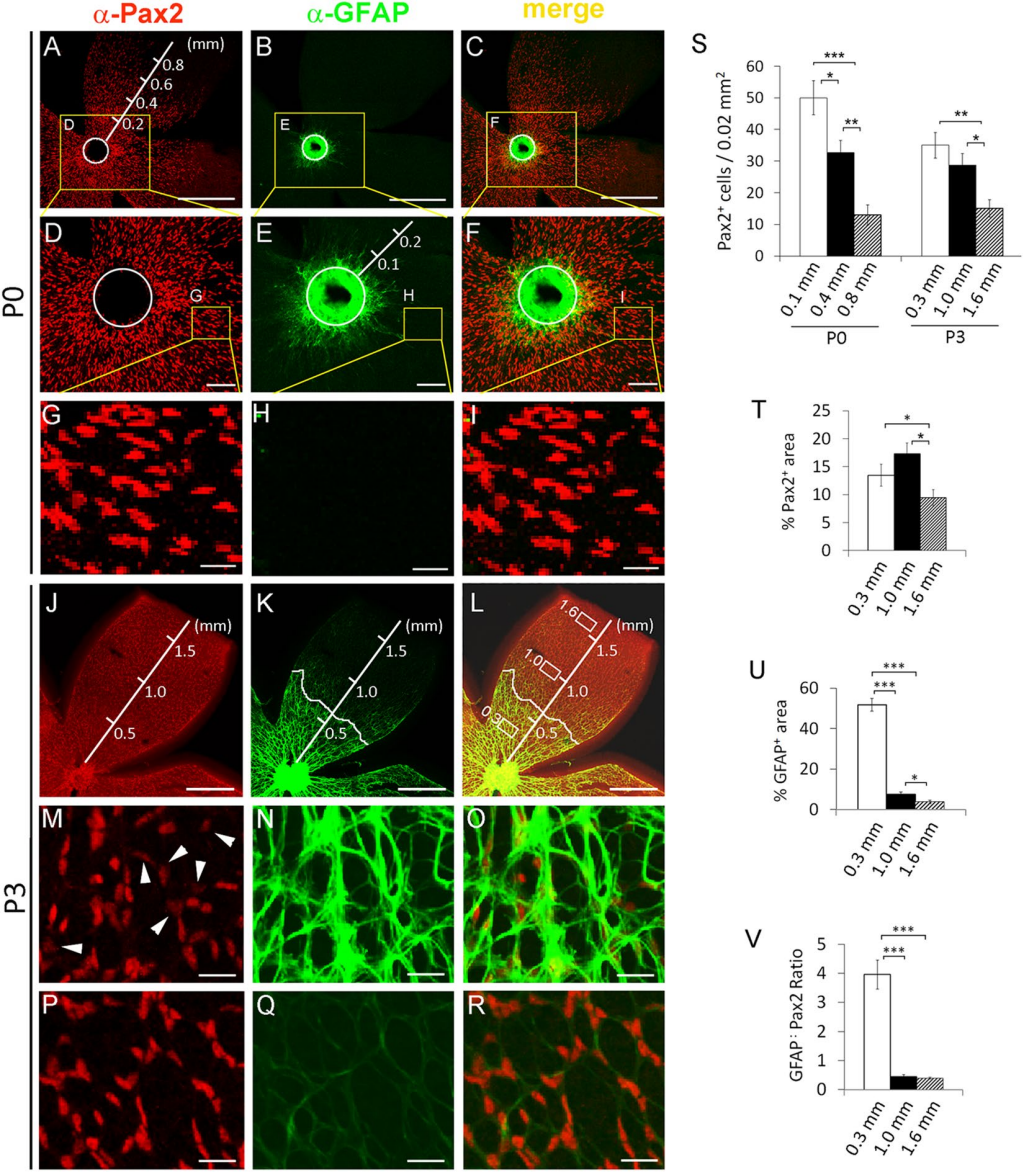
Retinal astrocytic development in early neonatal mice. Retinas were dissected from wild-type neonatal mice at P0 or P3, fixed, and subject to whole mount IF staining and flat-mounted confocal microscopy. (**A**–**I)**, retinas at P0. Circles in A to F trace the outer rim of the ONH. Co-localized Pax2^+^ and GFAP^+^ signals were present along the outer rim of the ONH and in the retinal areas immediately adjacent to it (**C** and **F**). Further away from the ONH, Pax2^+^ cells were generally GFAP^−^. GFAP^+^ cells of the ONH were mostly Pax2^−^. Outside the ONH, strong GFAP^+^ staining was visible within approximately 0.1 mm from the ONH outer rim, whereas weak GFAP^+^ signals extended to about 0.3 mm. (**J**–**R**), retinas at P3. J, Pax2^+^ cell population had expanded to near the retinal periphery. M and P, high magnification images for areas at 0.3 mm and 1.0 mm. White arrowheads in M point to weak Pax2^+^ signals. (**K** and **L**), strong GFAP^+^ signals were mostly present within 0.6 mm, whereas weak GFAP^+^ staining dominated areas further beyond. (**N** and **O**), areas at 0.3 mm; (**Q** and **R**), areas at 1.0 mm. The curvy white lines in **K** and **L** mark the border between GFAP^hi^ and GFAP^lo^ areas, and represent the astrocytic front. Note that GFAP^hi^ and GFAP^lo^ cells were both Pax2^+^. (**S**), number (#) of Pax2^+^ cells per 0.02 mm^2^ tissue area. Values below the horizontal axis indicate the distance between center of the area being quantified and the ONH outer rim; (**T**), % Pax2^+^ area, defined as % in a 0.02 mm^2^ tissue area occupied by Pax2^+^ signals; measured as percentage of a corresponding image area occupied by Pax2^+^ pixels; (**U**), % GFAP^+^ area; (**V**), GFAP:^+^Pax2 ratio ( = % GFAP^+^ area ÷ % Pax2^+^ area). n = 4. SEM values are shown. *p < 0.05; **p < 0.01; ***p < 0.001. Scale bars: A–C and J–L, 500 µm; D–F, 100 µm; G–I and M–R, 25 µm.

To clarify whether APCs and astrocytes existed in the retinas of newborn mice, we dissected retinas at P0 and performed whole-mount anti-Pax2 and anti-GFAP double immunofluorescence (IF) staining. As shown in flat-mount confocal images (Fig. 1A–I), strongly GFAP^+^ (GFAP^hi^) astrocytes were present in the ONH and in the retinal area within about 0.1 mm from the ONH, whereas weakly GFAP^+^ (GFAP^l^°) astrocytes could be found between approximately 0.1 and 0.3 mm from the ONH. APCs (Pax2^+^/GFAP^−^) were abundantly present within approximately 0.7 mm of the ONH (roughly halfway to the retinal periphery) (Fig. 1A–I). While some of the retinal Pax2^+^ cells were mature or immature astrocytes as indicated by their strong or weak GFAP expression, respectively, most of the Pax2^+^ cells were APCs because they were clearly GFAP^−^. APCs were located ahead of astrocytes, suggesting that they were migrating towards the retinal periphery. The population density of Pax2^+^ cells was visibly higher near the ONH and lower further away (Fig. 1A). This distribution pattern was confirmed by quantifying the number of Pax2^+^ nuclei per 0.02 mm^2^ tissue area at different retinal locations (Fig. 1S).

In contrast to the retinal astrocytes which were double positive for Pax2 and GFAP, the astrocytes tightly packed within the ONH were strongly GFAP^+^ but mostly Pax2^−^ except for some Pax2+ signals along the outer rim (Fig. 1D–F). The apparent co-localization of Pax2^+^ and GFAP^+^ signals along the outer rim probably reflected the overcrowding of APCs (Pax2^+^/GFAP^−^) and ONH astrocytes (Pax2^−^/GFAP^+^). Some of the co-localized signals might represent *bona fide* Pax2^+^/GFAP^+^ cells, especially in the area immediately surrounding the ONH (Fig. 1F and Supplemental Figure S1). It is unlikely that differentiated astrocytes migrated from the ONH into the retina, because the ONH astrocytes were mostly Pax2^−^ whereas retinal astrocytes were all Pax2^+^, although both were GFAP^+^. Instead, the presence of some retinal astrocytes at P0 was probably due to differentiation from APCs that had entered the retina ahead of time, via an intermediate step as IACs. Astrocytic differentiation might have occurred during the hours between the time the pups were born and when they were discovered and euthanized.

### Astrocytic precursors but not mature astrocytes led the way towards retinal periphery

We wondered if the expansion of the retinal astrocyte network entailed the migration of astrocytic precursors or differentiated astrocytes. Therefore, we further examined the spatial and temporal distribution patterns of these cells. At P1, the majority of GFAP^hi^ cells were located within about 0.2 mm of the ONH, whereas GFAP^l^° cells existed in a wide area between approximately 0.2 and 1.0 mm from the ONH (Supplemental Figure S2, A–F). In addition, APCs (Pax2^+^/GFAP^−^) were occasionally present at or near the migrating front (Supplemental Figure S2, F), which was typically at 0.9 to 1.1 mm from the ONH or 0.4 to 0.6 mm to the retinal periphery,.

At P3, GFAP^hi^ cells existed within approximately 0.6 mm of the ONH. GFAP^l^° had reached or almost reached the retinal periphery (approximately 1.55 to 1.85 mm from the ONH or 0 to 0.3 mm to the peripheral margin)(Fig. 1J–R, and Supplemental Figure S3). APCs (Pax2^+^/GFAP^−^) were occasionally present at or near the migrating front (Supplemental Figure S3), but not in more centrally located regions (Fig. 1P–R). Between GFAP^hi^ and GFAP^l^° regions, GFAP^+^ staining intensity changed sharply (Fig. 1K and L). Since strong GFAP expression is a hallmark of astrocytic differentiation, the line tracing along the border between GFAP^hi^ and GFAP^l^° regions represents the “astrocytic front” (Fig. 1K and L). Also note that retinal GFAP^+^ cells remained Pax2^+^ (Fig. 1M–R).

The above data indicated that APCs first occupied large areas of the retina, and then underwent partial differentiation to become IACs. The expansion of the network of differentiated astrocytes was due to progressive differentiation of prepopulated APCs and IACs in the radial direction, with the zone of active differentiation advancing from near the ONH at P0 towards the retinal periphery in the subsequent days. However, differentiated astrocytes themselves did not seem to spread or migrate towards the retinal periphery.

### Quantitative characterization of retinal astrocytic differentiation

To provide objective criteria for astrocytic differentiation, we quantified several parameters related to astrocytogenesis, including the number of Pax2^+^ cells per 0.02 mm^2^ tissue area in representative locations, percentage of an area occupied by GFAP^+^ pixels (% GFAP^+^ area), percentage of an area occupied by Pax2^+^ pixels (% Pax2^+^ area), and the ratio between the two (GFAP:Pax2). We focused on three representative areas (0.02 mm^2^ each) in P3 retinas: (1) an area at the center of the GFAP^hi^ domain; (2) an area at the center of the GFAP^l^° domain, and (3) an area at the migrating front. For simplicity, these areas will be referred to by their distances to the ONH, which were 0.3, 1.0, and 1.6 mm, respectively (Fig. 1K and L).

The number of Pax2^+^ cells was similar at 0.3 or 1.0 mm, but lower at 1.6 mm (Fig. 1S). The values for % Pax2^+^ area were also higher at 0.3 mm and 1.0 mm but lower at 1.6 mm (Fig. 1T). However, % GFAP^+^ area was different at all three locations, 51.7 ± 3.1% at 0.3 mm, 7.5 ± 1.1% at 1.0 mm, and 3.7 ± 0.8% at 1.6 mm (Fig. 1U). To provide a more reliable criterion for astrocytic differentiation, we calculated GFAP:Pax2 ratios for each area (GFAP:Pax2 = % GFAP^+^ area ÷ % Pax2^+^ area). As shown in Fig. 1V, the GFAP:Pax2 ratio were 3.95 ± 0.50 at 0.3 mm, 0.45 ± 0.06 at 1.0 mm, and 0.39 ± 0.03 at 1.6 mm. Thus, astrocytes at 0.3 mm were most differentiated among the three, whereas the other two areas were similarly immature.

### Pax2 expression was slowly but significantly downregulated after astrocytic maturation

**Figure 2:**
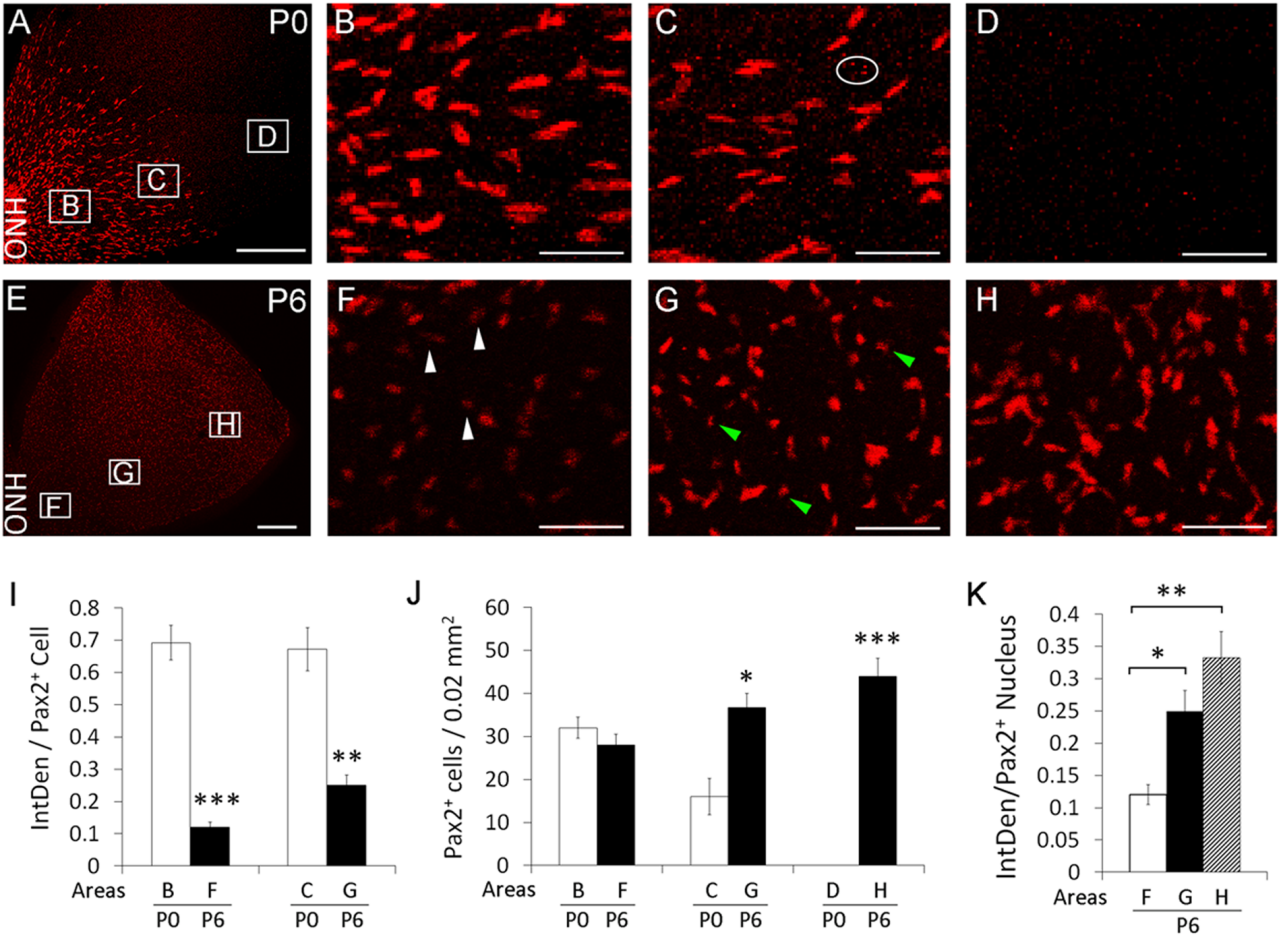
Downregulation of Pax2 expression in differentiated astrocytes. Retinas were isolated at P0 (**A**–**D**) and P6 (**E**–**H**), fixed, and subject to whole-mount anti-Pax2 IF staining and flat-mount confocal imaging. Lettered rectangles in A and E are shown at higher magnifications in panels with corresponding lettering. White arrowheads in F point to small and faintly stained Pax2^+^ nuclei. Green arrowheads in G indicate small nuclei with intermediate levels of Pax2^+^ signals. (**I**), comparison of IntDen between P0 and P6 retinas, but also note the similarity between the bars representing areas C and B of P0 retinas; (**J**), number of Pax2^+^ cells per 0.02 mm^2^ retinal tissue area; (**K**), IntDen at different areas of P6 retinas. Letters below the horizontal axes in (**I**–**K**) indicate areas where quantifications were carried out, and match the lettered rectangles in A or E. IntDen for a Pax2^+^ nucleus. Nonspecific speckles (such as those indicated by the oval in **C**) were excluded by setting lower size limit to 100 pixels. n = 4. *p < 0.05; **p < 0.01; ***p < 0.001. Error bars are SEM. Scale bars: A and E, 300 µm, all others, 50 µm.

At P0, retinas exhibited large and bright Pax2^+^ nuclei in the central half of the retina, with the more peripheral half remaining to be populated (Fig. 2A–D). By P6, Pax2^+^ nuclei appeared smaller and dimmer in the central half of the retina, especially near the ONH (Fig. 2E–H). However, Pax2 downregulation wasn’t instantaneous upon astrocytic differentiation, but instead progressed over a period of several days. For example, Pax2^+^ nuclei near the ONH were only moderately dimmer than their more peripheral counterparts at P3 (compare Fig. 1M and P), but much more so by P6 (compare Figure 2F and H).

To substantiate this observation, we measured integrative density (IntDen) of Pax2^+^ nuclei, defined as the product between the image area occupied by a Pax2^+^ nucleus (inch^2^) and mean grey value in the nucleus. Specifically, we calculated average IntDen for Pax2^+^ nuclei per 2 inch^2^ area in images, which equaled to 0.02 mm^2^ of actual tissue area. Near the ONH, IntDen was 0.69 ± 0.053 at P0 (Fig. 2B and I) but only 0.12 ± 0.015 at P6 (Fig. 2F and I), representing roughly a 5 fold difference. However, Pax2^+^ cells number at this location was similar between P0 and P6 (Fig. 2J), suggesting that Pax2 expression was not completely lost at P6. At about 0.6 mm from the ONH, the IntDen value was also higher at P0 although the difference was not as dramatic as near the ONH (Figure 2C,G,and I).

We also compared IntDen values between different areas in the same retina. At P0, Pax2^+^ IntDen values were similarly high near the ONH and at the migrating front (Fig. 2I). At P6, Pax2^+^ IntDen values were lower near the ONH but higher in more peripheral areas (Fig. 2K). Because Pax2^+^ cells near the ONH had existed as differentiated astrocytes for multiple days by P6, the low Pax2^+^ IntDen values of their nuclei were consistent with gradual Pax2 downregulation after astrocytic differentiation.

### Retinal vascular development slightly preceded astrocytic maturation

**Figure 3:**
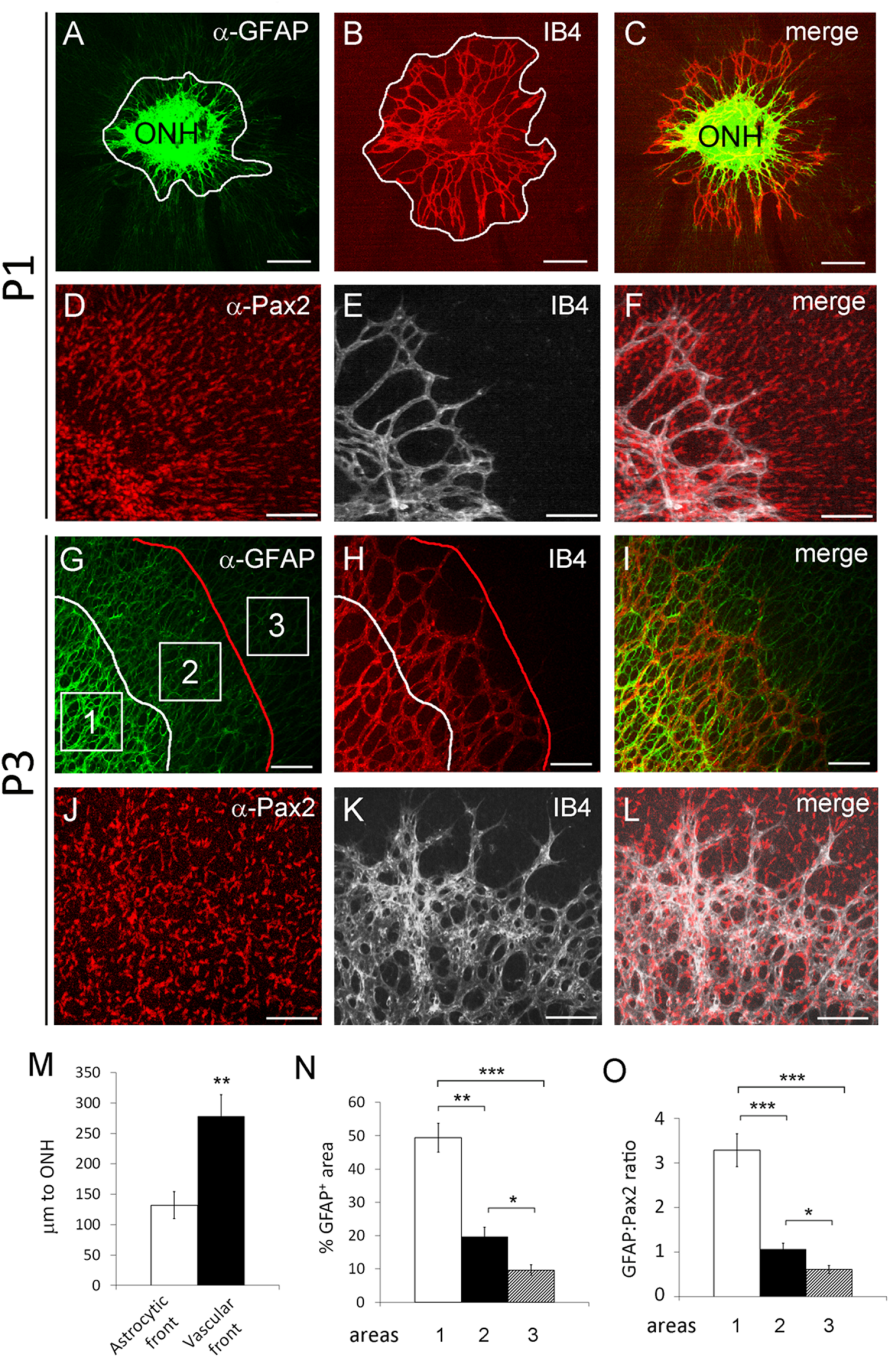
Relative positions of astrocytes and blood vessels in normal neonatal retinas at P3. Retinas were dissected at P1 and P3, fixed in 4% PFA, and subject to whole-mount IB_4_ or IF staining for Pax2 and GFAP expression. Flat-mount confocal images are shown in (**A**–**F**) (P1) and (**G**–**L**) (P3). Blood vessels were stained with IB_4_-Alexa Fluor® 594 (**B** and **H**) or IB_4_- Alexa Fluor® 647 (**E** and **K**). Curvy white lines in A and G mark the astrocytic front. The white line in (**B**) and the red line in (**H**) mark the vascular front. (**M**) distances between the astrocytic or vascular front and the edge of the ONH (µm to ONH). N and (**O**), quantification of astrocytic differentiation in areas 1, 2, and 3 (squares in G). n = 4. *p < 0.05; **p < 0.01; ***p < 0.001. Error bars are SEM. Scale bars: (**A**–**C**) and (**G**–**I**), 200 µm; (**D**–**F**) and (**J**–**L**), 100 µm.

The relative positions of mASCs, IACs, and blood vessels were studied by whole-mount IF and isolectin B4 (IB_4_) staining followed by confocal imaging of flat-mount retinas. At P1, the vascular front was located ahead of GFAP^hi^ but behind GFAP^l^° regions (Fig. 3A–C). While GFAP^hi^ signals were mostly present behind the vascular front, Pax2^+^ signals were abundantly present on both sides of the vascular front (Fig. 3D–F).

At P3, the retinal vascular front continued to advance towards the retinal periphery ahead of the astrocytic front but behind most of the GFAP^lo^ cells (Fig. 3G–I), except for a narrow band of GFAP^lo^ cells between the vascular front and the astrocytic front (Fig. 3G, between the white and red lines). GFAP^+^ signals in this area appeared somewhat stronger than those ahead of the vascular front but still substantially weaker than in the GFAP^hi^ area behind the astrocytic front. Regardless their relative positions to the vascular front or astrocytic front, all GFAP^+^ cells were Pax2^+^ (Fig. 3J–L,and Supplemental Figure S4). However, not all Pax2^+^ cells were GFAP^+^. Although low in abundance, a few Pax2^+^ cells remained GFAP^−^ near the periphery of P3 retinas (Supplemental Figure S4).

To determine if the relative positioning of the vascular front and astrocytic front was statistically significant, we compared their distances to the ONH in P1 retinas. As shown in Fig. 3M, the vascular front was 278 ± 36 µm from the outer rim of the ONH, whereas the corresponding value for the astrocytic front was only 132 ± 22 µm. The difference was statistically significant (p < 0.01). At P3, we quantified astrocytic differentiation at three locations, behind the astrocytic front (area 1), between astrocytic and vascular fronts (area 2), and in front of the vascular front (area 3) (Fig. 3N and O). Area 1 was by far the most differentiated of all three (GFAP:Pax2 ratio of 3.3 ± 0.37), area 2 was much less differentiated than area 1 (GFAP:Pax2 ratio of 1.06 +/− 0.13), and area 3 was least differentiated (GFAP:Pax2 ratio of 0.61 ± 0.09). Overall, the difference between areas 2 and 3 was modest, but both were very different from area 1. The astrocytes in area 2 were newly exposed to blood vessels, and were most likely just beginning to differentiate towards mASCs.

### Retinal vascular development was essential for astrocytic differentiation

**Figure 4:**
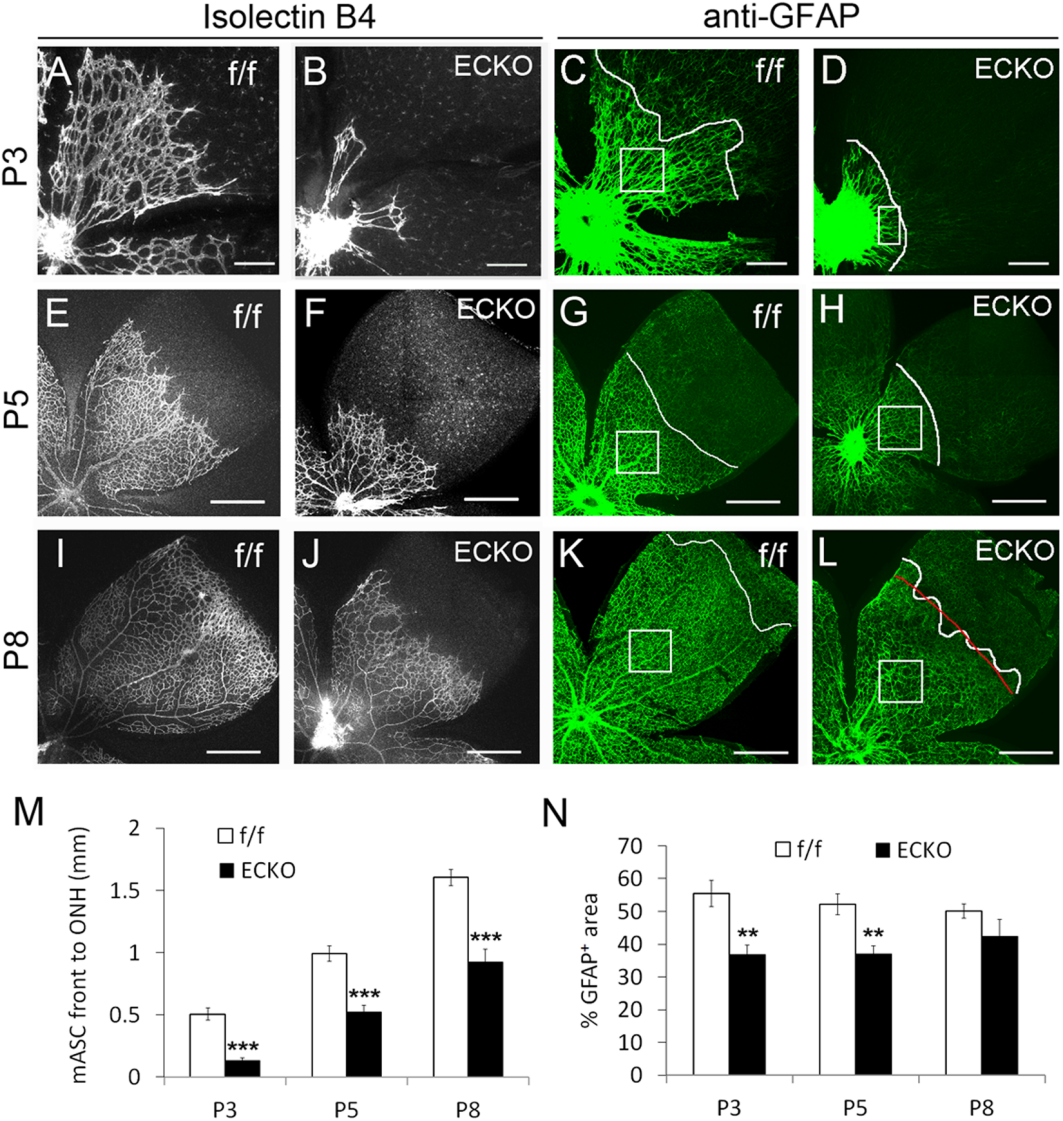
Dual deficiency in retinal vascular development and astrocytic differentiation in *Vegfr-2* ECKO mice. *Vegfr-2* ^flox/flox^ (f/f) and *Vegfr-2* ^flox/flox^/Cdh5(Pac)^CreERT2^ (ECKO) mice received tamoxifen by daily oral gavage between P0 and P2. Retinas were dissected at P3, P5, and P8, fixed in 4% paraformaldehyde, and subject to whole-mount staining with IB_4_-Alexa® 594 (**A**,**B**,**E**,**F**,**I**, and **J**) and rat anti-GFAP/goat anti-rat IgG-Alexa Fluor®-488 (**C**,**D**,**G**,**H**,**K**, and **L**). Stained retinas were flat-mounted, and imaged by laser confocal microscopy. Curvy white lines in GFAP^+^ images mark the astrocytic front. M, quantification of distance (mm) between the ONH outer rim and astrocytic front. Where the white line tracing the astrocytic front was too zigzag to determine its distance to the ONH, a red line was drawn through its estimated average position when NIH ImageJ-assisted measurements were taken, with one such example shown here (**L**). N, % GFAP^+^ area within mature astrocyte-occupied regions. Quantifications were carried out in representative areas (white squares in **C**,**G**,**H**,**K**, and **L** or rectangle in **D**). n = 6. **p < 0.01, ***p < 0.001. Errors are SEM. Scale bars: (**A**–**D**), 200 µm; (**E**–**L**), 500 µm.

To investigate the role of retinal angiogenesis in astrocytic differentiation, we disrupted retinal angiogenesis by endothelial cell (EC) specific *Vegfr-2* targeting. Cdh5(Pac)^CreERT2^ transgenic mice used in this study had demonstrated efficiency and EC specificity in neonatal mouse retinas^29^, which was confirmed in our own hands (Supplemental Figure S5, A–F). Floxed *Vegfr-2* mice^30,31^ were crossed with Cdh5(Pac)^CreERT2^ transgenic mice, and EC specific *Vegfr-2* disruption was accomplished by treating *Vegfr-2* ^flox/flox^/Cdh5(Pac)^CreERT2^ mice with daily oral gavage of tamoxifen at P0–P2. The resulting *Vegfr-2* ECKO mice survived for 6–9 days, whereas floxed mice treated in parallel remained healthy. At P3, P5, and P8, retinal vascular development was severely compromised in *Vegfr-2* ECKO mice but appeared normal in floxed mice (Fig. 4A,B,E,F,I and J, and Supplemental Figure S6). Importantly, retinal astrocytic development was also severely defective in *Vegfr-2* ECKO mice, displaying shorter distances between the astrocytic front and the ONH at all three stages examined (Fig. 4C,D,G,H,K,L and M). Even between the ONH and the astrocytic front, % GFAP^+^ area values were significantly lower in *Vegfr-2* ECKO mice at P3 and P5 (Fig. 4N, and Supplemental Figure S7). Overall, retinal astrocytic differentiation was considerably slowed but not completely stopped by vascular deficiency. As a control, astrocytogenesis appeared normal in tamoxifen-treated Ai9/Cdh5(Pac)^CreERT2^ tdTomato reporter mice (Supplemental Figure S5, G–R).

### Accumulation of IACs and APCs in Vegfr-2 ECKO retinas

**Figure 5:**
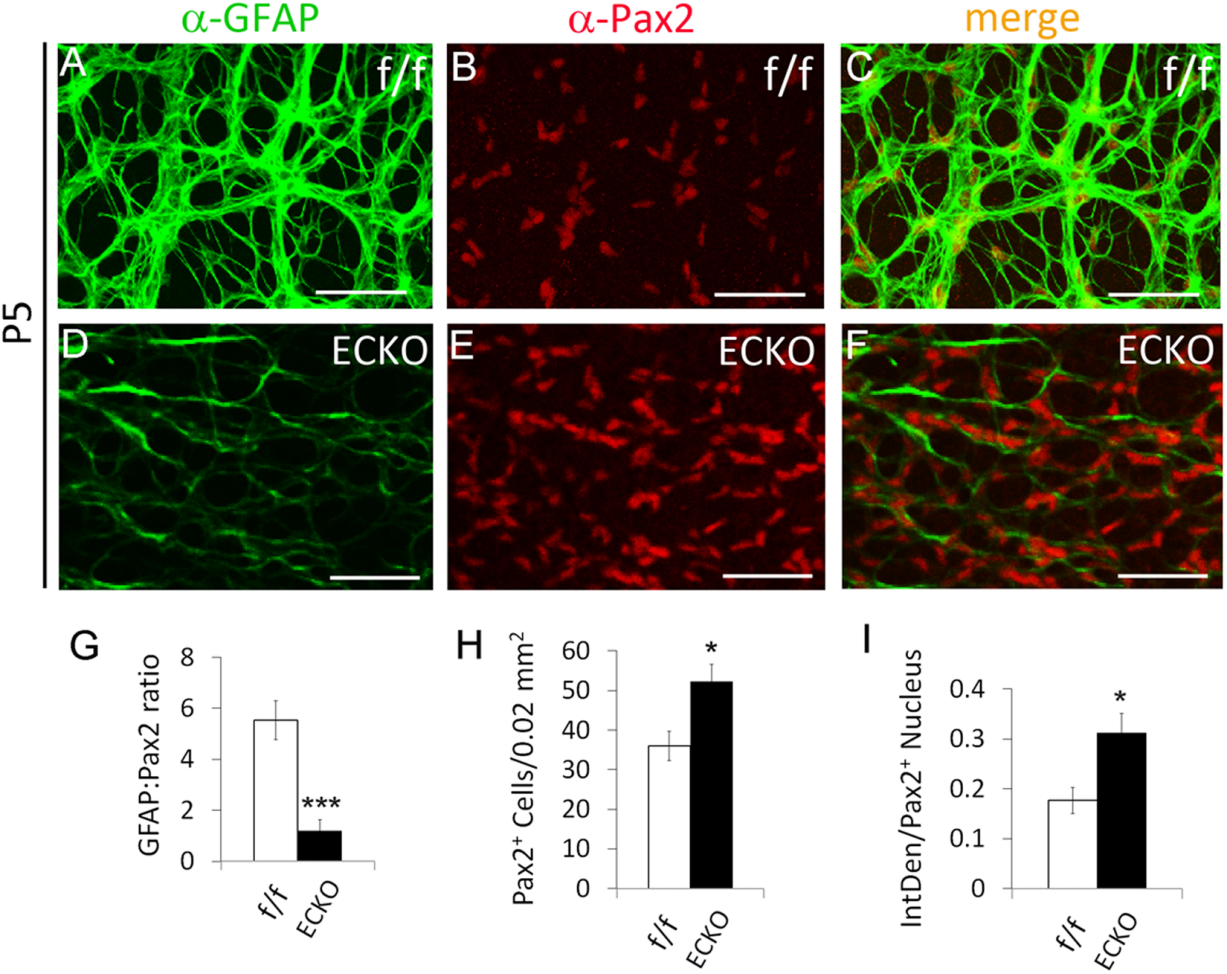
Accumulation of immature retinal astrocytes (Pax2^+^/GFAP^lo^) in *Vegfr-2* ECKO mice. Retinas were isolated at P5 from floxed and *Vegfr-2* ECKO mice, fixed in 4% PFA, and incubated first with anti-Pax2 and anti-GFAP and then secondary antibodies. Stained retinas were flat-mounted and imaged by confocal microscopy. (**A**–**F**) images from areas at approximately 0.7 mm from the ONH. (**G**–**I**) GFAP:Pax2 ratio (**G**), number of Pax2^+^ cells per 0.02 mm^2^ tissue area (**H**), and IntDen values per Pax2^+^ nucleus (**I**). n = 6. *p < 0.05; ***p < 0.001. Errors bars are SEM. Scale bars: 50 µm.

**Figure 6:**
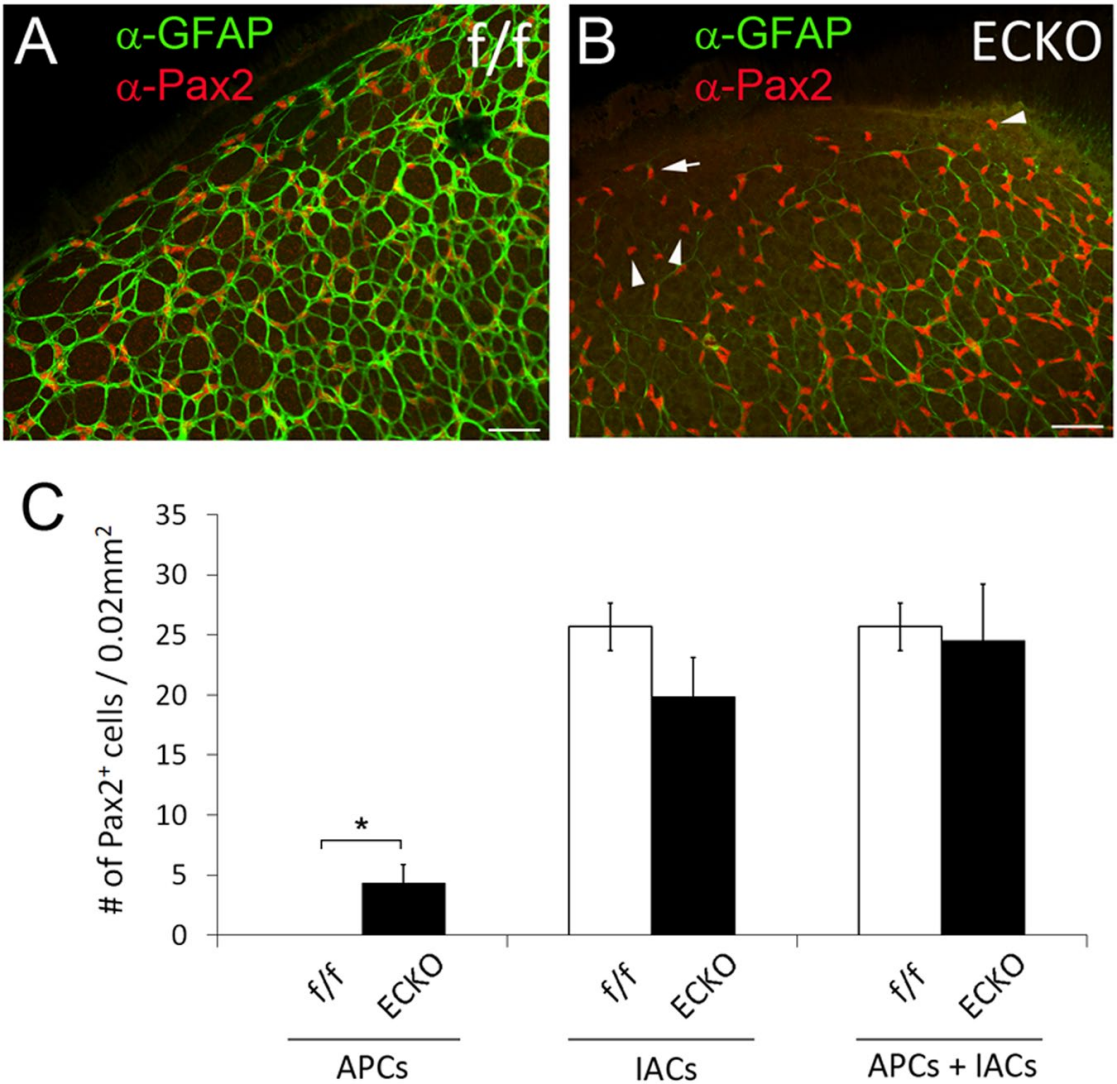
Accumulation of APCs (Pax2^+^/GFAP^−^) near the retinal periphery of *Vegfr-2* ECKO mice. Retinas were dissected from tamoxifen treated *Vegfr-2* ^flox/flox^ (f/f) and *Vegfr-2* ^flox/flox^/Chd5(Pac)^CreERT2^ (ECKO) mice, fixed, and stained by anti-Pax2 and anti-GFAP and appropriate secondary antibodies. Confocal images were taken under the condition to maximize the visibility of weak GFAP^+^ signals. A and B, retinal peripheral areas. In floxed mice (**A**), all Pax2^+^ cells were GFAP^+^, but in *Vegfr-2* ECKO mice, many Pax2^+^ cells remained GFAP^−^ (**B**), arrowheads point to some examples). In addition, many other Pax2^+^ cells had just trace amounts of GFAP^+^ staining (B, the arrow points to an example). (**C**) quantification of Pax2^+^ cells, including Pax2^+^/GFAP^−^ cells, Pax2^+^/GFAP^+^ cells, and the sum of both. Quantification was carried out in 0.1 mm × 0.2 mm peripheral tissue areas. For each retina, quantification was carried out in four different peripheral areas, and the average was used as one data point. n = 6. *p < 0.05. Errors bars are SEM. Scale bars were 50 µm.

We further examined retinal astrocytogenesis at P5, focusing on two locations: (1) at about 0.7 mm from the ONH (Fig. 5), and (2) near the retinal periphery (Fig. 6). At 0.7 mm, floxed mice displayed GFAP^hi^ staining and moderate Pax2^+^ staining (Fig. 5A–C). The GFAP:Pax2 ratio was 5.53 ± 0.76 (Fig. 5G), indicating advanced differentiation. By contrast, *Vegfr-2* ECKO mice mostly had GFAP^lo^ cells in this area but brighter and larger Pax2^+^ nuclei, with an average GFAP:Pax2 ratio of 1.18 ± 0.46 (Fig. 5D–G), demonstrating the immature nature of astrocytes in this area. *Vegfr-2* ECKO mice had both increased number of Pax2^+^ cells per 0.02 mm^2^ (Fig. 5H) and higher IntDen values (Fig. 5I). We also quantified APCs (Pax2^+^/GFAP^−^) at the retinal periphery. Per 0.02 mm^2^ tissue area, the floxed mice had 0 APC whereas *Vegfr-2* ECKO mice had 4.3 ± 1.5 (Fig. 6). Taken together, the above data indicated that astrocytic precursors, mostly IACs but also to some extent APCs, accumulated in *Vegfr-2* ECKO retinas.

**Figure 7:**
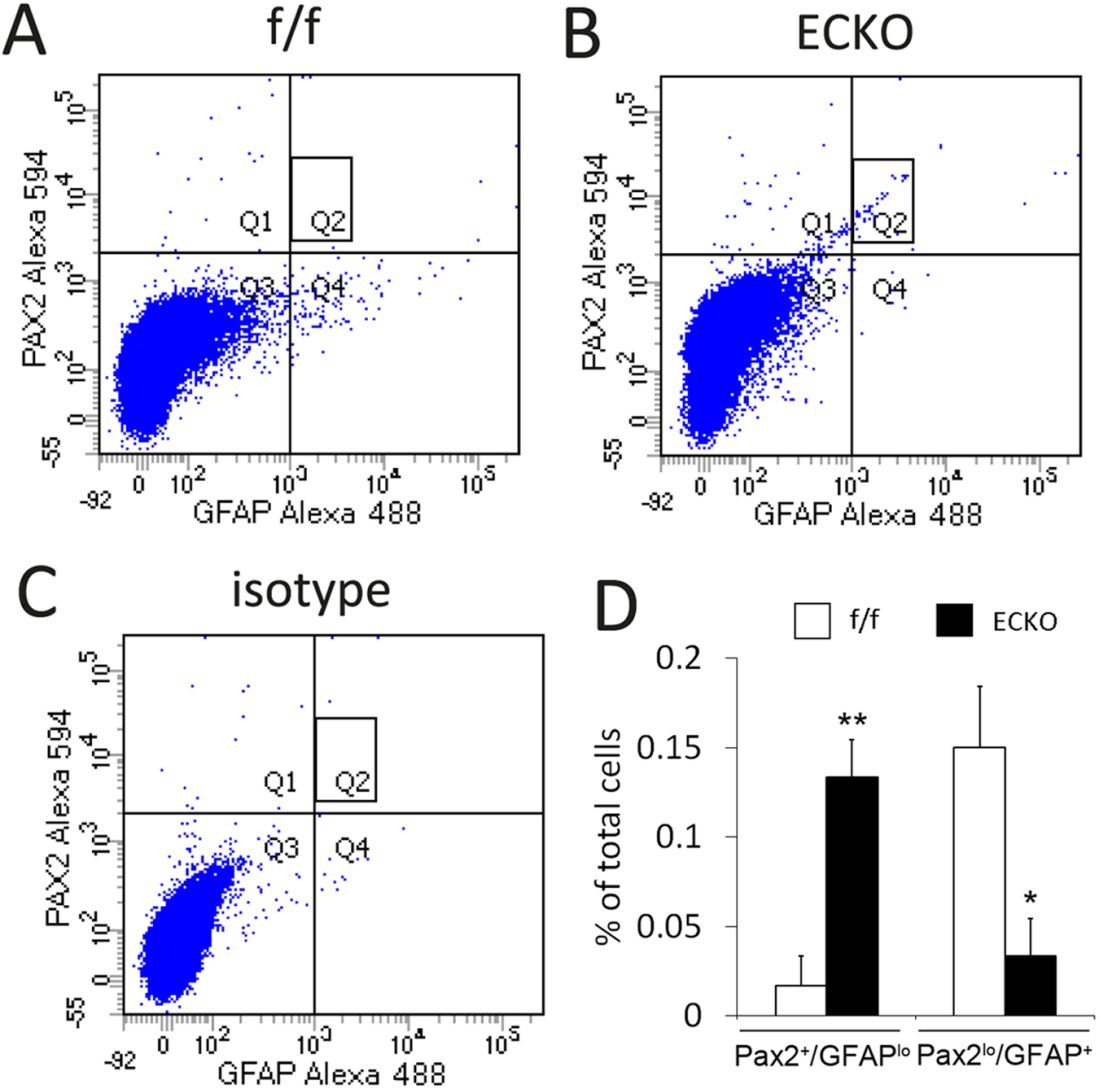
Quantification of astrocytic differentiation in floxed (f/f) and *Vegfr-2* ECKO mice by flow cytometry. Single cell suspensions were prepared from P5 retinas, briefly fixed and permeabilized. Cells were then incubated with rabbit anti-mouse Pax2, rat anti-mouse GFAP, or isotype controls, followed by goat anti-rabbit IgG-Alexa Fluor® 594 and goat anti-rat IgG-Alexa Fluor® 488. Stained cells were washed, and subject to flow cytometry analysis. (**A**) Floxed mice (f/f); (**B**), Vegfr-2 ECKO mice (**C**) an example of isotype control using cells from floxed mice. For quantification, background events in isotype controls were subtracted from corresponding values in floxed or Vegfr-2 ECKO samples. Quantification of Pax2^+^/GFAP^lo^ (Q1) and GFAP^+^/Pax2^lo^ events (Q4) are presented in D. Also note a subpopulation of cells in *Vegfr-2* ECKO mice exhibiting intermediate staining intensities for both Pax2 and GFAP (**B**, rectangle in Q2). Similar cells were virtually absent in floxed mice (**A**, rectangle in Q2). *p < 0.05, **p < 0.01. Errors are shown as SEM.

To complement the confocal imaging-based analyses, we performed flow cytometry analysis (Fig. 7). Single cell suspensions from P5 retinas were stained with anti-Pax2 and anti-GFAP followed by fluorophore conjugated secondary antibodies. Flow cytometry analysis indicated that relative to floxed mice, *Vegfr-2* ECKO mice had five-fold more of IACs (GFAP^lo^/Pax2^+^, Q1) but three-fold less of differentiated astrocytes (GFAP^+^/Pax2^lo^, Q4). In addition, the *Vegfr-2* ECKO mice contained a population of cells with intermediate staining intensities for both GFAP and Pax2 (rectangle in Q2). Such cells were essentially absent in floxed mice. The exact nature of this subpopulation is unclear but they may reflect the accumulation of cells at intermediate stages of differentiation. The corresponding subpopulation of intermediately stained cells have not been definitively identified in confocal images yet, but note that a proportion of GFAP^+^ cells in *Vegfr-2* ECKO mice did seem to display more modest intensity than those in floxed mice (for example, see Supplemental Figure S7, C and D). In short, the flow cytometry data indicated that progression from precursors to differentiated astrocytes was suppressed in *Vegfr-2* ECKO mice.

**Figure 8:**
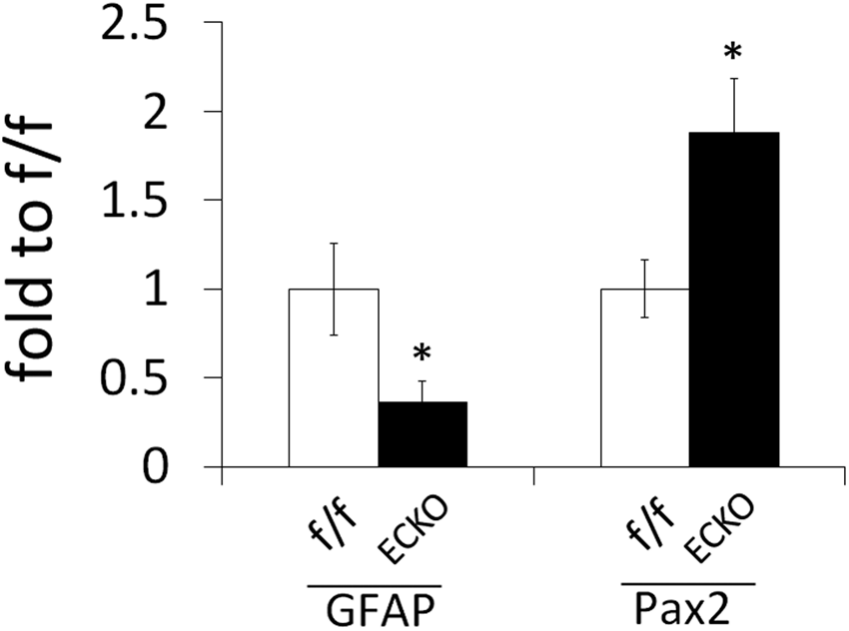
Quantification of GFAP and Pax2 expression. qPCR was performed for total RNA samples isolated from PDGFRα^+^ cells in P5 retinas. qPCR signal values for GFAP and Pax2 were normalized to β-actin, and further calculated as fold to floxed mice (f/f). n = 8 mice. *p < 0.05, Error bars are SEM.

The expression levels of GFAP and Pax2 were also quantified by quantitative RT-PCR (qPCR). Retinal astrocytes were isolated as PDGFRα^+^ cells by fluorescence activated cell sorting, and total RNA was isolated and subject to qPCR. Data in Fig. 8 indicate that in *Vegfr-2* ECKO mice, astrocytic GFAP expression was downregulated whereas Pax2 expression was upregulated, consistent with less mature status of retinal astrocytes in VEGFR2 deficient mice.

Because astrocytic precursors are proliferative whereas mature astrocytes are largely quiescent, we assessed whether *Vegfr-2* ECKO retinas contained more proliferative Pax2^+^ cells. Specifically, we labelled proliferative cells by intraperitoneal injection of 5′-Bromo-2′deoxy-Uridine (BrdU) at P3, and visualized Pax2^+^/BrdU^+^ cells by whole-mount IF staining with anti-Pax2 and anti-BrdU antibodies. Quantification was carried out in small areas at approximately 0.3 mm from the ONH. This area was chosen because earlier experiments had shown that at P3, astrocytes at this location were already mature in floxed mice but not in *Vegfr-2* ECKO mice (Fig. 4C and D), thus making it suitable for analyzing whether delayed maturation was associated with increased proliferation. Consistent with their less differentiated states, Pax2^+^ cells in *Vegfr-2* ECKO retinas were more proliferative than in floxed controls, reflected by the presence of more Pax2^+^/BrdU^+^ cells than in floxed controls (Supplemental Figure S8).

### Oxygen exposure or APC specific HIF-2α deficiency promoted astrocytic differentiation

**Figure 9:**
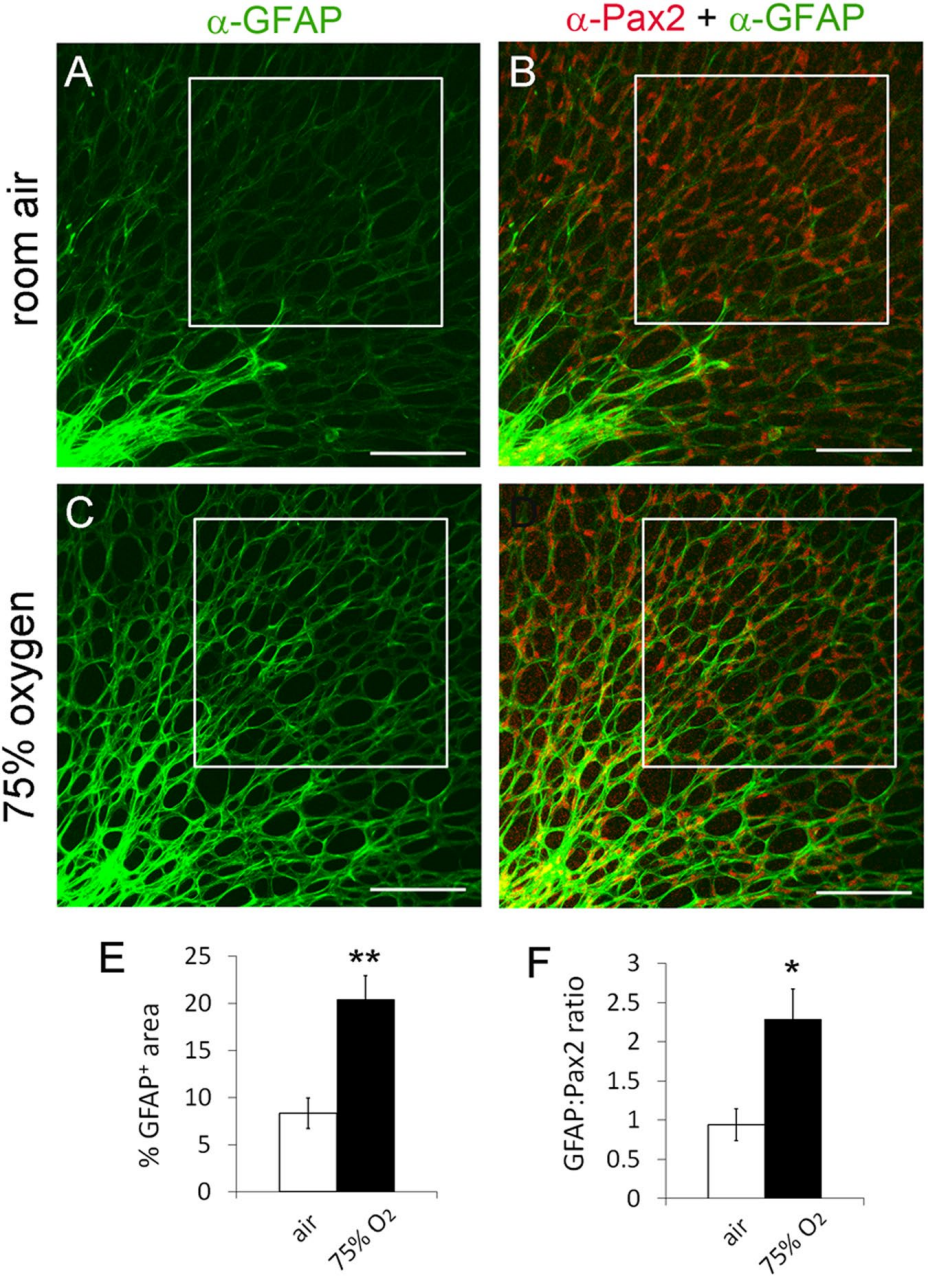
Acceleration of retinal astrocytic differentiation by oxygen. At P0, wild-type CD1 mice were either maintained under room air (**A**) or placed into an oxygen chamber (75% O_2_) (**B**). After 24 hours, mice were euthanized, and retinas were dissected, fixed, and stained as whole-mounts by anti-Pax2 and anti-GFAP and fluorophore conjugated secondary antibodies. Stained retinas were flat-mounted and imaged by confocal microscopy. Astrocyte differentiation in boxed areas was quantified, and results are summarized in (**E** and **F**). n = 6 mice. *p < 0.05, **p < 0.01. Error bars are SEM. Scale bars are 100 µm.

To test the effect of oxygen on retinal astrocytic differentiation, we exposed newborn wild-type mice to 75% oxygen for 24 hours. For quantification, we focused on small squared areas with their closest points at about 0.2 mm off the ONH. This location was selected for analysis because it was next in line to undergo astrocytic differentiation in normal P1 retinas. Thus, if hyperoxia accelerated astrocytic differentiation the consequence would be first revealed at this location. As shown in Fig. 9A–D, GFAP^+^ signals appeared to be much stronger in oxygen-treated mice. Quantitatively, % GFAP^+^ area values in the specified squares were 8.35 ± 1.6% in room air controls and 20.4 ± 2.53% in oxygen-treated mice, respectively; GFAP:Pax2 ratios were 0.94 ± 0.20 under room air and 2.29 ± 0.38 under hyperoxia (Fig. 9E and F). These data demonstrated that retinal astrocytic differentiation advanced much further in hyperoxia-exposed mice.

**Figure 10:**
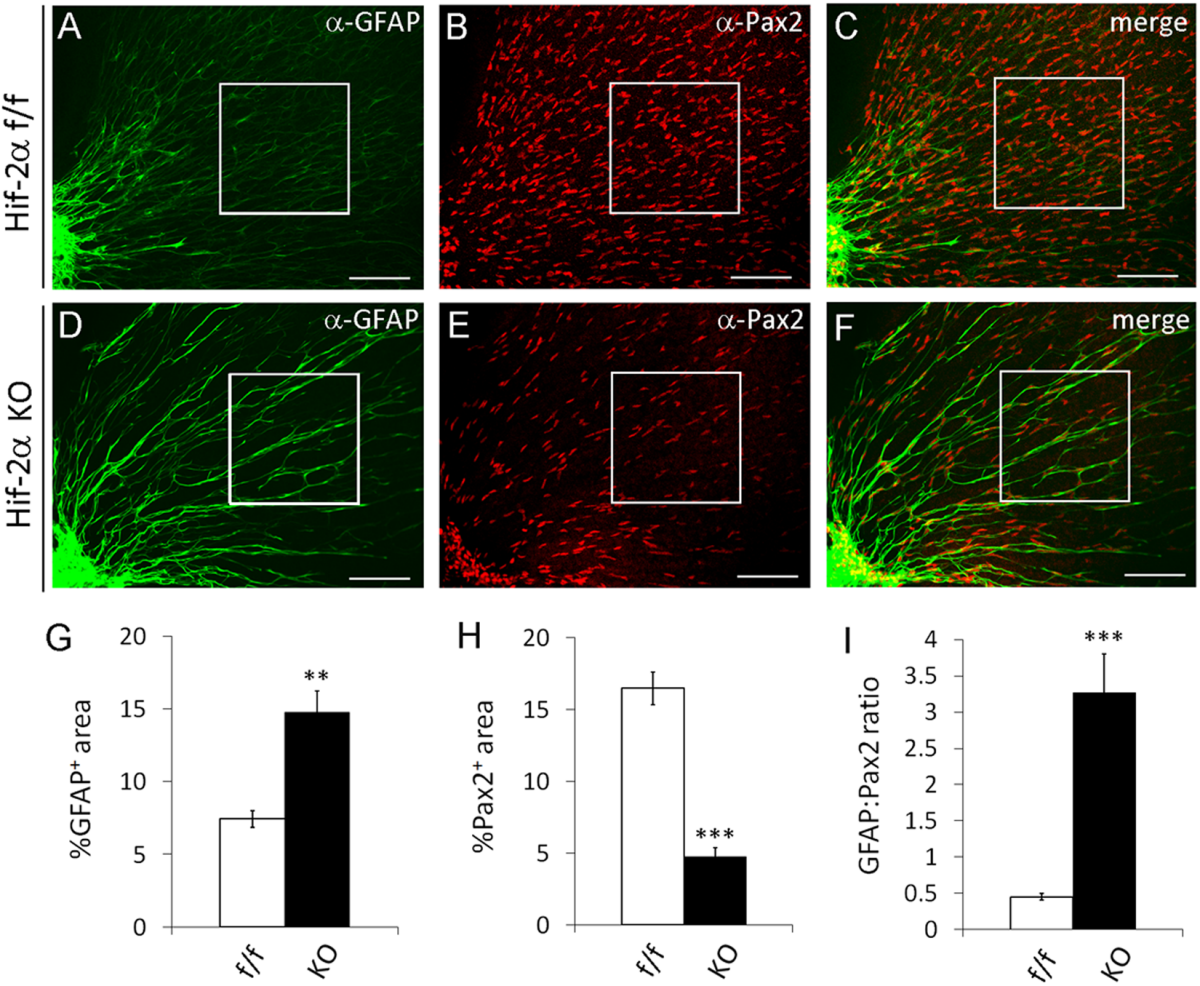
Accelerated retinal astrocytic differentiation in APC specific *Hif-2α* knockout mice. Retinas were isolated from *Hif-2α* ^*flox/flox*^ (f/f) and *Hif-2α* ^*flox/flox*^/GFAPCre (KO) mice at P1. After whole-mount staining with anti-Pax2 and anti-GFAP and fluorophore conjugated secondary antibodies, retinas were flat mounted and imaged by confocal microscopy. Boxed areas were quantified for astrocytic differentiation, and data are shown in (**G**,**H**, and **I**). n = 5 mice. **p < 0.01, ***p < 0.001. Error bars represent SEM. Scale bars are 100 µm.

The effects of oxygen on retinal astrocytic differentiation were qualitatively related to retinal astrocytic phenotypes in *Hif-2α* ^*flox/flox*^/GFAPCre mice, in which GFAPCre had demonstrated activity in APCs^32,33^. By contrast, astrocytic development in *Hif-1α* ^*flox/flox*^/GFAPCre mice was normal. Therefore, we further examined astrocytic differentiation in *Hif-2α* ^*flox/flox*^/GFAPCre mice (Fig. 10A–F). At P1, HIF-2α deficient mice had higher % GFAP^+^ area (Fig. 10G), lower % Pax2^+^ area (Fig. 10H), and higher GFAP:Pax2 ratio (Fig. 10I) than their floxed controls. Given that retinal angiogenesis does not begin until birth, at the stage of analysis (P1) there were only negligible amounts of retinal blood vessels in all mice (Figure S9). Accelerated astrocytic differentiation due to loss of HIF-2α suggested that in normal development, oxygen diffusing out blood vessels might induce astrocytic differentiation in part by triggering HIF-2α degradation in nearby astrocytic precursors.

## Discussion

At the normal morphological level, much of the knowledge about retinal astrocytogenesis was based on studies in rats^5,6,34^. Optic nerve-derived APCs provide the source for retinal astrocytic development, and begin to migrate into the retina as early as embryonic day (E) 15. GFAP^+^ cells begin to appear around the ONH at E16, populating the central third of the inner retinal surface by P0 and reaching the retinal periphery by P9–P10. Much less is known about the morphological events of retinal astrocytic development in normal mice, especially in the critical early neonatal days.

Therefore, we examined retinal astrocytic morphogenesis in normal neonatal mice. At P0, APCs were numerous in the central half of the inner retinal surface, most of which differentiated into IACs by P1. Both APCs and IACs were migratory although there were only a few APCs remaining after P1. Thus, IACs were mostly responsible for prepopulating the retinal inner surface ahead of astrocytic differentiation. IACs reached the peripheral margin as early as P3, and the latest by P5. Our finding that immature astrocytes reached the peripheral margin at such an early time point does not conflict with the literature which typically states that the astrocyte network reaches the peripheral margin between P8 and P10, because in the literature the phrase “astrocyte network” usually refers to the network of mature astrocytes (mASCs). In our studies, the network of mASCs reached the periphery at similar ages as described in the literature. However, our data indicated that the network of mASCs expanded not by migration but by differentiation from prepopulated IACs.

We concluded that the network of differentiated astrocytes did not serve as a template for angiogenesis. Instead, its own formation was dependent on angiogenesis. A key finding supporting this conclusion was that retinal astrocytic differentiation was suppressed in vessel-deficient retinas of EC specific *Vegfr-2* knockout mice. Our findings are consistent with a recent study showing that chemical inhibition of VEGF receptor signaling delayed both retinal vascularization and astrocyte network formation^35^. However, the availability of EC specific *Vegfr-2* knockout mice enabled us to unequivocally establish a cause and effect relationship between poor retinal vascular development and defective astrocytic differentiation. Furthermore, we found that retinal blood vessels were required not only for IAC to mASC differentiation, but also for APCs to undergo partial differentiation and become IACs, even though APCs were located far away from blood vessels. Thus, it seemed that a diffusible molecule or molecules, rather than physical contacts between blood vessels and astrocytic precursors, were important for differentiation.

Oxygen may be one of the diffusible molecules mediating the role of blood vessels in astrocytic differentiation. This possibility is supported by our observation that hyperoxia significantly accelerated astrocytic differentiation. Because it was reported that long-term (several days) hyperoxia exposure led to retinal hypoxia as a result of failed retinal angiogenesis^36,37^, we limited oxygen exposure to only 24 hours. Under this condition, hyperoxia-exposed mice displayed more advanced astrocytic differentiation than their room air counterparts. Oxygen probably induced astrocytic differentiation by causing HIF-2α degradation. Faster astrocytic differentiation due to APC specific HIF-2α knockout provided direct support to this possibility. It should be pointed out however, that faster differentiation in HIF-2α deficient mice ultimately led to less number of astrocytes after the initial increase, because astrocytic differentiation in these mice was accelerated to a point to cause rapid depletion of the APC pool, thus prematurely terminating astrocytic development^32^.

Our finding that astrocyte network formation depends on retinal vascular development represents an apparent conflict with the longstanding astrocyte template theory. However, the two can be reconciled by specifying that the phrase “astrocyte template” refers to immature astrocytes but not differentiated astrocytes. In other words, it will be helpful to state explicitly that retinal vascular development is guided by a preexisting network of immature astrocytes (instead of a preexisting network of astrocytes). Extending from this rephrased astrocyte template theory, we hypothesize that possums lack astrocytic and vascular networks because they lack retinal APCs to begin with, resulting in the subsequent lack of immature astrocytes which are essential for both angiogenesis and subsequent development of a differentiated astrocyte network. A similar proposition may also explain simultaneous deficiencies in retinal vascular and astrocyte networks in several knockout mouse models^8–11^.

**Figure 11:**
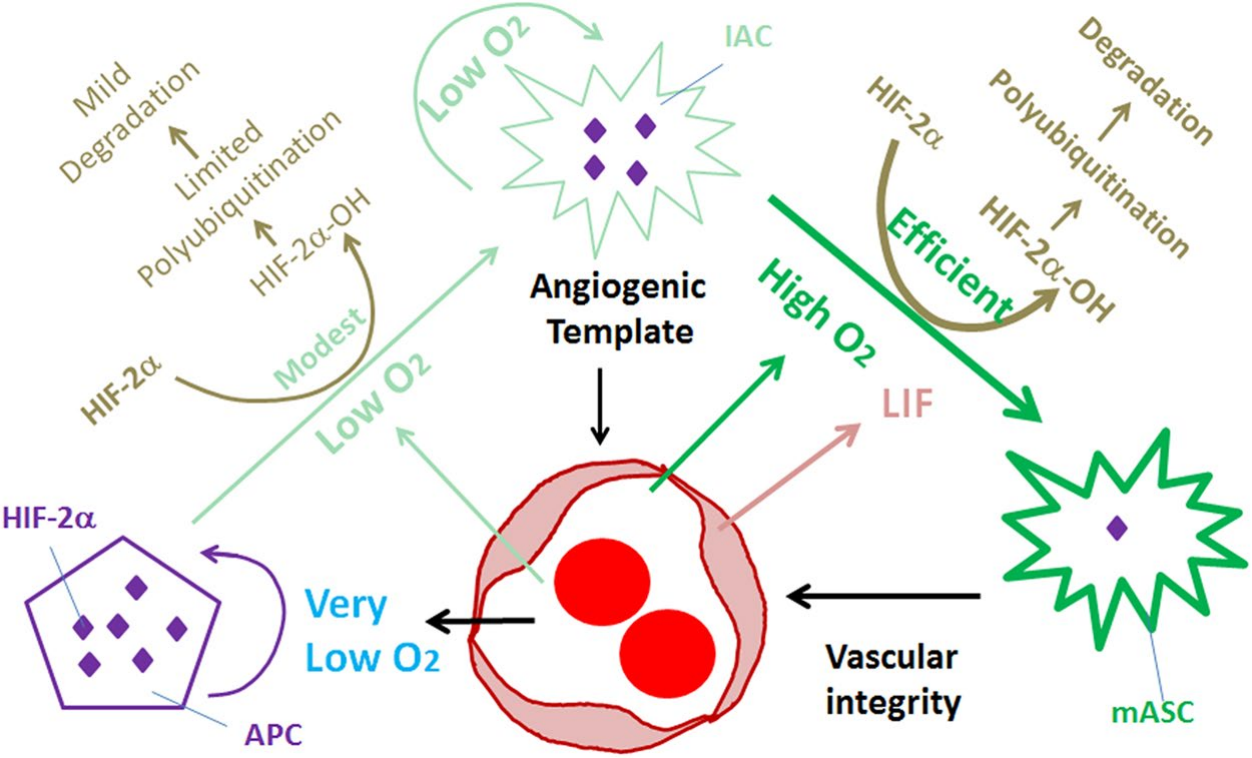
Interdependence between retinal astrocytic and vascular development in neonatal mice. APCs may be severely hypoxic due to their remote locations from vascularized areas. Hypoxia favors the accumulation of HIF-2α (depicted as many purple diamonds), which in turn promotes APC status^32^. IACs may be under varying degrees of hypoxia depending on their distances from vascularized areas, but the extent of hypoxia may be generally lighter than in APCs (except in a small number of IACs located more peripherally than APCs). Intermediate levels of oxygen may permit HIF-2α hydroxylation at modest efficiencies. Ongoing synthesis and degradation may result in a steady state level of HIF-2α that is lower than in APCs but higher than in mASCs (represented by the relative numbers of purple diamonds). mASCs are well oxygenated by being within vascularized areas. High levels of oxygen promote efficient HIF-2α prolyl hydroxylation and fast degradation. However, mASCs probably still retain trace amounts of HIF-2α which are functionally important (depicted as a single purple diamond). This possibility is supported by the fact that *Hif-2α* knockout in differentiated astrocytes led to compromised retinal vascular stability^46^. Besides oxygen, LIF is also known to induce astrocytic differentiation from immature astrocytes^11,34,44^. With regard to angiogenesis, IACs but probably not APCs are critical, due to the remote distances of APCs from the vascular front. The responsible angiogenic factor(s) are unknown, but IACs might facilitate angiogenesis by providing a physical surface for endothelial cells to adhere and migrate over^7^.

Integrating data reported in this article with those published earlier by us and others^11,32^, a working model may be proposed to help future studies on this subject (Fig. 11). Optic nerve-derived APCs prepopulate large areas of inner retinal surface at perinatal stages, differentiating into IACs and continuing to proliferate and migrate. As they migrate towards the retinal periphery, IACs support angiogenesis behind them, likely by mechanisms that require fibronectin but not VEGF-A^38,39^. APC to IAC differentiation occurs at locations far away from blood vessels but IAC to mASC differentiation occurs only within vascularized areas, suggesting that low levels of oxygen are adequate for APC to IAC differentiation but higher levels are required for IAC to mASC differentiation. Oxygen may promote astrocytic differentiation by inducing HIF-2α prolyl hydroxylation, a biochemical reaction in which O_2_ is used as a substrate by prolyl hydroxylase domain proteins to hydroxylate specific prolyl residues in HIF-α proteins, labeling them for rapid polyubiquitination and proteasomal degradation^32,40–43^. Besides oxygen, EC-derived leukemia inhibitory factor (LIF) may also promote retinal astrocytic differentiation^11,34,44^. Once fully differentiated, astrocytes no longer promote angiogenesis but may provide support to retinal vascular integrity. This latter function still requires HIF-2α and VEGF-A^39,45,46^, implying that normal mASCs still have residual levels of HIF-2α and VEGF-A despite being in well oxygenated microenvironments.

The implications of this study are twofold. First, while the ECs have been found to secrete protein factors to regulate cell differentiation, to the best of our knowledge this article may be the first to report that vascular oxygenation function also has a regulatory role in cell differentiation. Second, a role of the retinal vasculature in astrocytic differentiation complements the astrocyte template theory. It may be stated that while the network of immature astrocytes supports angiogenesis, the differentiated astrocyte network is the product of blood vessel-dependent astrocytic differentiation, rather than a template to induce angiogenesis. In conclusion, the retinal vascular and astrocytic components shape each other’s development through reciprocal regulatory mechanisms.

## Materials and Methods

### Mice

Animal protocols were approved by the Institutional Animal Care and Use Committee at the University of Connecticut Health Center in compliance with Animal Welfare Assurance. Floxed *Vegfr-2* mice were generated by the Sato lab as previously described^3^. Cdh5(Pac)^CreERT2^ mice were kindly provided by Ralf Adams^29^. Ai9 mice were from the Jackson Laboratories (stock numbers 007909), and carried a Cre-dependent tdTomato reporter transgene^47^. GFAPCre transgenic mice carried a Cre transgene under the control of human GFAP promoter (Jax stock number 004600)^33^. CD1 mice were purchased from Charles River; C57BL/6 mice were purchased from the Jackson Laboratories. Wild-type or genetically modified mice were bred to a mixed strain background of 25% CD1 and 75% C57BL/6, unless specified in particular experiments. Mouse colonies were maintained on normal chow with a 12 light/12 darkness cycle. Mice were bred by natural mating, and neonatal mice were designated postnatal day 0 (P0) in the morning following their birth. For studies requiring analyses at P0, newborn litters were visually inspected in the morning for strong pinkish skin coloring.

### Genotyping

Mouse toe clips (before P8) or ear punch pieces were used for genotyping. PCR primers for the *Vegfr-2* locus were FlkFlox1 (TS967) 5′-AGGAGCCAGGCATTCTCCAAG-3′; Flklox2 (TS1975) 5′ CCACAGAACAACTCAGGGCTA-3′ and Flklox3 (TS1976) 5′-GGGAGCAAAGTCTCTGGAAA-3′. Product sizes were 231 base pair (bp) for the floxed allele, 185 bp for the wild-type allele, and 600 bp for the deleted allele. Primers for the Chd5(Pac)^CreERT2^ transgene included Cdh5CreF2 (5′TGGAGCCAGGCGGATAGTGAG3′) and Cdh5CreR2 (5′GCCTGGCGATCCCTGAACAT3′), 560 bp for the Cre transgene; no band without the transgene. PCR primers and band lengths for the *Hif-2α* locus and GFAPCre transgene were described previously^32^. PCR reactions were carried out using Taq DNA polymerase (Life Technologies) as follows: 95 °C for 9 minutes for 1 cycle; then 94 °C for 0.5 minute, 55 °C for 1 minute, 72 °C for 4 minutes, 35 cycles.

### Immunofluorescence (IF) and isolectin B_4_ (IB_4_) staining of whole-mount retinas

Retina isolation and staining were carried out as previously described^32,48^ but are briefly summarized below. Eyes were isolated from euthanized neonatal mice by enucleation, and immediately fixed in 4% paraformaldehyde (PFA) (45 minutes, room temperature). Subsequently, retinas were isolated, and four incomplete radial incisions were made at roughly equal spacing, generating four petals attached to one another near the ONH. Retinas were first blocked overnight in Retina Staining Buffer (RSB, consisting of phosphate buffered saline, 1 mM CaCl_2_, 1 mM MgCl_2_, 1% Triton × 100, and 1% BSA), and subsequently incubated with antibodies or isolectin B_4_ (IB_4_) in RSB. Antibodies included rabbit anti-mouse Pax2 (1 µg/ml, Life Technologies), rat anti-mouse GFAP (2 µg/ml, Life Technologies), goat anti-rat IgG-Alexa Fluor® 488 (1:250, Life Technologies), and donkey anti-Rabbit IgG-Cy3 (1:400, Jackson ImmunoResearch, West Grove, PA). Vascular structures were stained with IB_4_-Alexa Fluor® 594 (5 µg/ml, Life Technologies), IB_4_-Alexa Fluor® 647 (5 µg/ml Life Technologies), or IB_4_-Alexa Fluor® 488 (5 µg/ml, Life Technologies) as indicated where appropriate. Stained retinas were washed 3 times in RSB (30 minutes each with rocking), and flat-mounted in 50% glycerol.

### Laser confocal microscopy

Stained and flat-mounted retinas were imaged by laser confocal microscopy. For Pax2^+^, IB_4_^+^, and BrdU^+^ signals, imaging conditions were set to optimize signal detection in a normal retinal specimen and then applied to all others in the same analysis. To image GFAP^+^ structures, imaging conditions were set up to balance the need of avoiding overexposure in the strongly GFAP^+^ central areas and detecting the weak GFAP^+^ signals at more peripheral locations. Unless specified, images were taken under such conditions. In some experiments, images were taken to achieve optimal sensitivity for weak GFAP^+^ signals at more peripheral areas. Maximal sensitivity was necessary to detect traces of GFAP^+^ signals so that weakly GFAP^+^ IACs would not be mistaken as GFAP^−^ APCs.

### BrdU labeling and quantification of BrdU^+^ cells

Proliferating cells were labeled with 5′-Bromo-2-deoxyuridine (BrdU, 120 mg/kg of body mass; Roche Applied Science, Indianapolis, IN) by intraperitoneal injection. At 1 hour after injection, neonatal mice were euthanized, and eyes were isolated by enucleation. Whole eyes were fixed in 4% paraformaldehyde and stored in 70% ethanol overnight. Retinas were then separated from other eye structures, incubated first in 1% Triton X-100 in PBS (30 minutes at room temperature) and then in 2 mol/L HCl for 1 hour at 37 °C. Processed retinas were double stained with mouse anti-BrdU (biotin) (Abcam) and rabbit anti-Pax2. Primary antibodies were detected by avidin-Alexa Fluor®−594 (Jackson ImmunoResearch, West Grove, PA) and goat anti-rabbit IgG-Alexa Fluor®−488, respectively. BrdU^+^ and BrdU^+^/Pax2^+^ cells were counted using the “multi-point” tool of the NIH ImageJ program.

### Flow cytometry

Neonatal retinas were isolated without fixation and minced into small pieces. Retinal pieces were submerged in Type 1 collagenase solutions (1 mg/ml in Serum-free DMEM, Gibco-Thermo Fisher Scientific), and digested for 45 minutes at 37 °C. Clumps were disrupted by pipetting up and down several times during digestion. Cell pellets were collected by centrifugation for 5 minutes at 1000 g. After carefully removing about 90% of the supernatant, pellets were resuspended in collagenase solutions to repeat the digestion step. Samples were then centrifuged for 5 minutes at 1200 rpm. Pellets were rinsed and resuspended in 0.025% trypsin, and incubated for 2 minutes at 37 °C. Digestion was stopped by the addition of DMEM containing 10% FBS, and cells were filtered through cell strainer (45 µm)(Thermo Fisher Scientific). Filtered cells were fixed in 2% PFA for 5 minutes with rocking, and permeabilized with 0.1% Triton X-100 in PBS. Fixed and permeabilized retinal cells were double stained with anti-Pax2 and anti-GFAP (or isotype controls) for 1 hour in staining buffer (PBS containing 1% BSA), washed several times, and then stained with secondary antibodies including goat anti-rabbit IgG-Alexa Fluor® 594 and goat anti-rat IgG-Alexa Fluor® 488. Stained cells were washed in PBS for three times by low speed centrifugation and resuspension. Stained cells were then subject to flow cytometry analysis.

### Quantitative PCR (qPCR)

Retinal single cell suspensions were prepared from P5 mice similarly as described under “Flow cytometry” section, except that the fixation step was omitted. Cells were incubated with anti-PDGFRα for 1 hour at 4 °C (1 µg/ml) followed by Alexa Fluor®−488-conjugated secondary antibody. Stained cells were washed by centrifugation (1000 g, 5 minutes), and used for fluorescence activated cell sorting (FACS) within the same day. Total RNA was isolated from PDGFRα^+^ cells using TRIzol method (Life Technologies). After reverse transcription using random hexamer primers, qPCR was performed using SYBR Green PCR master mix (Life Technologies). Primers were as follows: GFAP: 5′-CGGAGACGCATCACCTCTG-3′ and 5′-AGGGAGTGGAGGAGTCATTCG-3′; Pax2: 5′-AAGCCCGGAGTGATTGGTG-3′ and GTTCTGTCGCTTGTATTCGGC; β-actin: 5′-GGCTGTATTCCCCTCCATCG-3′ and 5-CCAGTTGGTAACAATGCCATGT-3′.

### Hyperoxia treatment

Newborn pups from the same litter were divided into two groups, with one group being placed in a 75% oxygen chamber and the other remaining in the room air. Foster mothers were used as necessary. After 24 hours, neonatal mice were euthanized. Retinas were isolated, fixed, and stained as descried under the section for IF and IB_4_ staining.

### Measurements of distances from the astrocytic or vascular front to the ONH

The astrocytic front was marked with a white line along the border between strongly and weakly GFAP^+^ areas, and the distance between the line and ONH outer rim was measured with the aid of NIH ImageJ. In cases where the white line was too zigzag for measurements, a red line was drawn along the estimated average position of the white line, and the distance between the red line and the ONH was measured. For retinas at P1, the astrocytic front was typically too zigzag to estimate average positions. For these retinas, the total area within the zigzag curve was measured using NIH ImageJ, and converted to the radius (R_1_) for a hypothetical circle of equal area size, using the mathematical formula A = πr^2^. The radius of the ONH was calculated similarly (R_2_). The distance between the astrocytic front and the ONH outer rim was D = R_1_ − R_2_. The distance between the vascular front and ONH was measured similarly.

### Quantification of Pax2^+^, GFAP^+^, and IB_4_^+^ signals in confocal images

Pax2^+^, GFAP^+^, and IB_4_^+^ signals were quantified using the NIH ImageJ software. % Pax2^+^ area was quantified as percentage area in a specified rectangle or square occupied by Pax2^+^ pixels. Background levels were determined by sampling several empty areas between Pax2^+^ nuclei. Data presented for % Pax2^+^ area represent the net value after subtracting the average background. % GFAP^+^ area was quantified similarly. However, % IB_4_^+^ area was calculated as the percentage area in an entire retinal petal occupied by IB_4_^+^ pixels. GFAP:Pax2 ratios = % GFAP^+^ area ÷ % Pax2^+^ area.

Pax2^+^ cell numbers were quantified with the aid of the “analyze particles” tool within the NIH ImageJ software. Nonspecific speckles were generally much smaller than the smallest Pax2^+^ nuclei in the same image, and were excluded from quantification by setting appropriate lower size limit. Integrated Density (IntDen) was defined as the product of the area size of a Pax2^+^ nucleus as it appears in the image and the corresponding mean grey value. Pax2^+^ IntDen values were quantified as the average per Pax2^+^ nuclei in specified image areas corresponding to 0.02 mm^2^ of tissue size.

### Statistical analysis

Student’s *t* tests were used to evaluate statistical significance. Data are presented as means ± standard errors of the mean (SEM). All sample sizes (n) refer to the number of mice analyzed per group. *p* < 0.05 was considered significant.

## Electronic supplementary material

Supplementary Information
