# Supplementary material for: Retinal Angiogenesis Regulates Astrocytic Differentiation in Neonatal Mouse Retinas by Oxygen Dependent Mechanisms
Source: Sci Rep. 2017 Dec 14;7:17608. doi: 10.1038/s41598-017-17962-2 (PMC5730567; doi:10.1038/s41598-017-17962-2)
Supplement: Supplementary file 1 — Supplementary Information [file 41598_2017_17962_MOESM1_ESM.pdf]

Retinal Angiogenesis Regulates Astrocytic Differentiation in Neonatal Mouse Retinas

by Oxygen Dependent Mechanisms

Li-Juan Duan, Sarah J. Pan, Thomas N. Sato, and Guo-Hua Fong

Supplemental Information

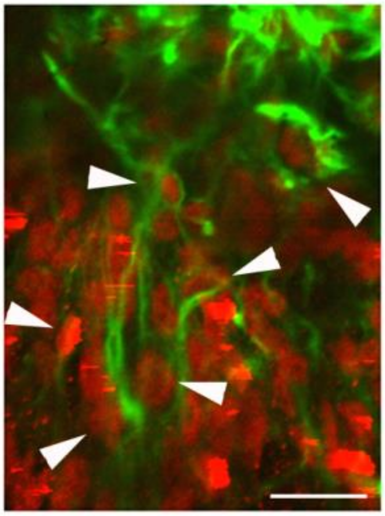

Figure S1. Pax2 and GFAP expression in retinal astrocytes at P0. The area near the ONH is shown at a high magnification. This area contains a mixture of Pax2$^+$/GFAP$^+$ (arrow heads) and Pax2$^+$/GFAP$^-$ cells. The scale bar is 20 µm.

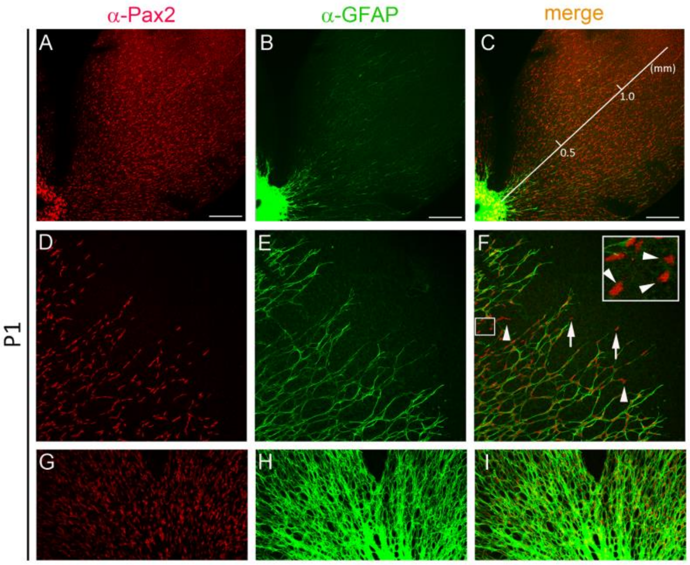

Figure S2. Astrocytic differentiation at P1. A-C, Confocal images of anti-Pax2 and anti-GFAP IF stained flat-mount retinas. The scaled line in C marks distance (mm) from the edge of ONH. D-F, the migrating front of the migrating Pax2$^+$ cell population, located at approximately 0.9 to 1.1 mm from the ONH. The total distance from the ONH to retinal periphery is about 1.5 mm at P1 (not shown). D-F, the migrating front of astrocytic precursors. In F, Inset was expanded from the boxed area; arrowheads in both the inset and the main image point to examples of APCs (Pax2$^+$/GFAP$^-$). Arrows point to examples of IACs that were just beginning to emerge from APCs (Pax2$^+$ cells exhibiting traces of GFAP$^+$ signals). Although some IACs were located ahead of APCs, in the scale of the entire retina such IACs accounted for <1%. Thus APCs are generally ahead of IACs with a few exceptions. G-I, an area near the ONH, included to show that the imaging condition offering most sensitive detection of GFAP$^+$ signals at the migrating front (E) results in overexposure near in central retinal areas (H). Scale bars: 500 µm for A-C, and 100 µm for D-I.

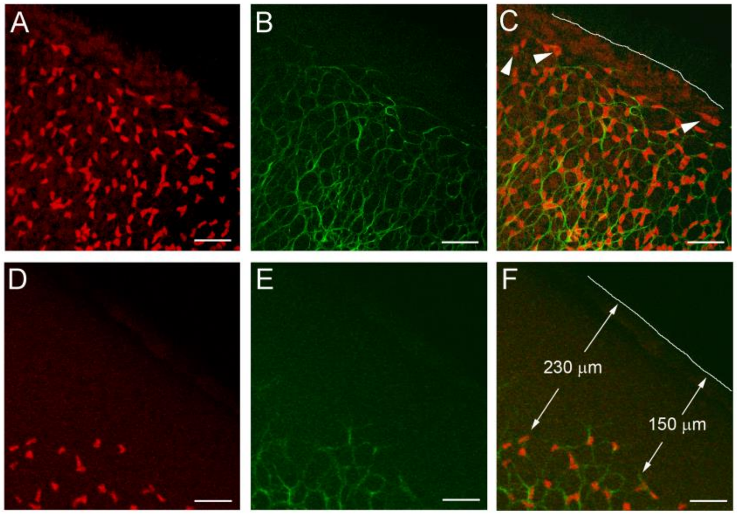

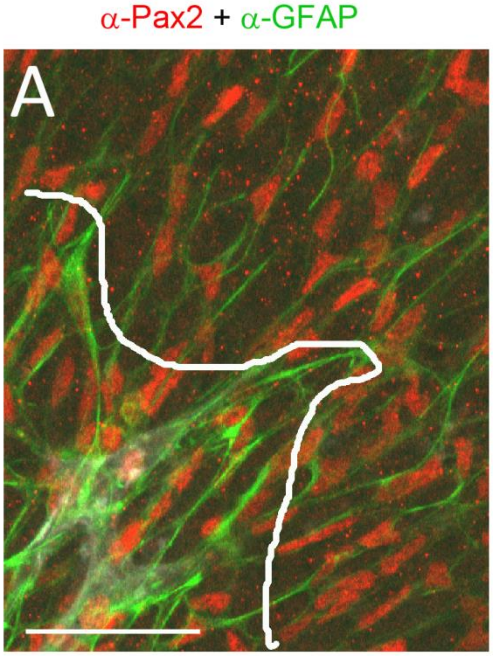

Figure S4. Co-expression of Pax2 and GFAP in immature and mature astrocytes. The confocal image shows an area spanning the area around the astrocytic front (curvy white line). Note the close association between Pax2^{+} nuclei and GFAP^{+} cytoskeletal fibers. Precise superimposition of the two signals were not observed, because Pax2^{+} signals were located in the nuclei whereas GFAP^{+} signals were in the cytoskeletal filaments. Scale bar is 50 µm.

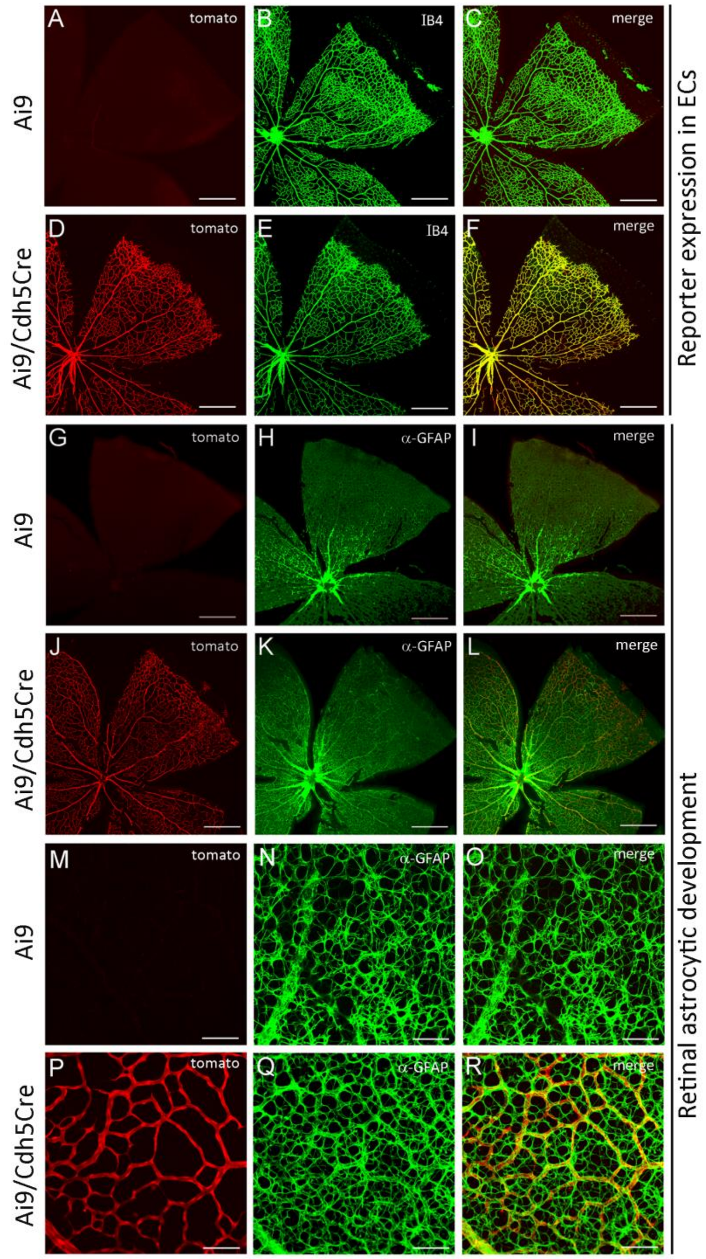

Figure S5. Efficiency and specificity of Cdh5(Pac)Cre^{ERT2}. To evaluate Cre specficity and efficiency, Cdh5(Pac)Cre^{ERT2} mice were crossed with Ai9 Cre-dependent tdTomato reporter mice, in which the tdTomato reporter transgene was preceded by floxed 3x SV40 polyA. Ai9 and Ai9/Cdh5(Pac)^{CreERT2} mice were both treated with tamoxifen by daily oral gavage between P0-P2. Retinas were dissected at P7 and stained with either isolectin B_4 (IB_4)-Alexa Fluor® 488 or rat anti-mouse GFAP followed by goat anti-rat IgG Alexa Fluor® 488. Stained retinas were flat-mounted and imaged for tdTomato expression or staining signals by confocal microscopy. Scale bars are 500 µm for A-L, 50 µm for M-R.

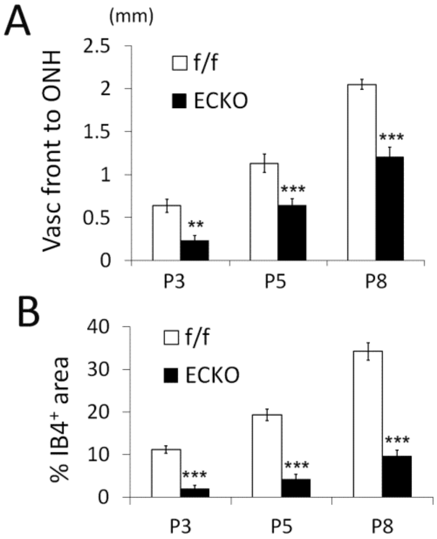

Figure S6. Quantification of retinal vascular development in floxed (f/f) and *Vegfr-2* ECKO mice. Flat-mount confocal images were taken from retinas stained with isolectin B_4-Alexa Fluor® 594, and quantified with the aid of the NIH ImageJ software. A, distance between the vascular front to the edge of ONH. B, % IB_4^+ area, referring to the percentage of the entire area in a petal occupied by IB_4^+ pixels (instead of % in a selected area).

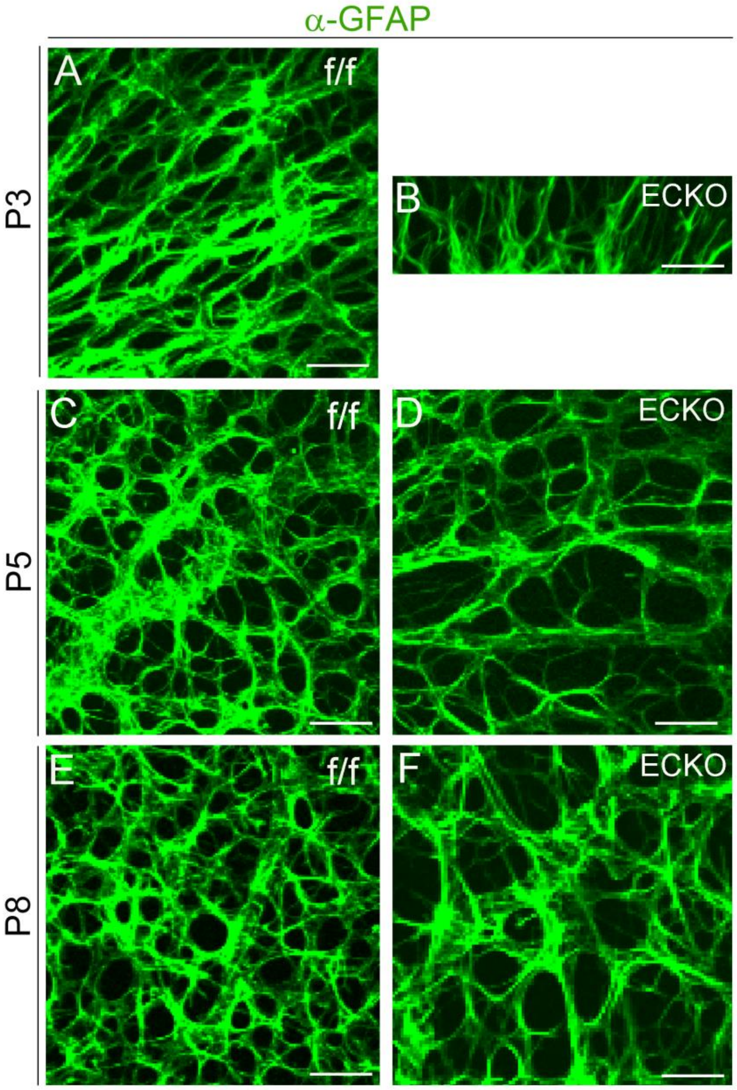

Figure S7. Astrocytic development in floxed (f/f) and *Vegfr-2* ECKO mice shown at higher magnification. Images are from boxed areas in Figure 4C, D, G, H, K, and L. Scale bars are 50 μm.

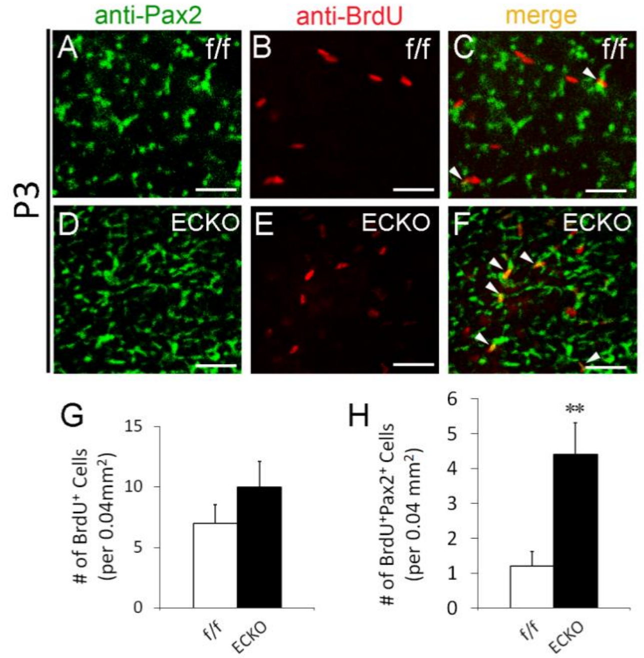

Figure S8. Proliferation assay of Pax2$^+$ cells by *in vivo* BrdU incorporation. *Vegfr-2$^{flox/flox}$* (f/f) and *Vegfr-2* ECKO neonatal mice were injected with BrdU at P3. An hour later, retinas were isolated, fixed, and incubated with anti-BrdU-biotin and rabbit anti-Pax2. Secondary reagents were avidin-Alexa Fluor® 594 and goat anti-rabbit Fluor® 488. Stained retinas were flat-mounted and analyzed by confocal microscopy. White arrowheads point to BrdU$^+$/Pax2$^+$ cells. **, $p < 0.01$. Scale bars are 50 µm.

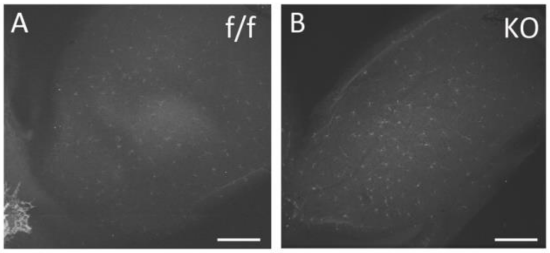

Figure S9. Retinal angiogenesis in P1 mice. A. Hif-2α$^{f/f}$ (f/f); B. Hif-2α$^{f/f}$/GFAPCre (KO). Flat mount P1 retinas were stained with IB4 and imaged by confocal microscopy. Scale bar is 200
